# Human brain transcriptome analysis finds region- and subject-specific expression signatures of GABA_A_R subunits

**DOI:** 10.1038/s42003-019-0413-7

**Published:** 2019-05-01

**Authors:** Adolfo Sequeira, Kevin Shen, Assaf Gottlieb, Agenor Limon

**Affiliations:** 1 Department of Psychiatry and Human Behavior, School of Medicine, University of California Irvine, Irvine, CA USA; 2 Department of Neurology, Mitchel Center for Neurodegenerative Diseases, School of Medicine, University of Texas Medical Branch, Galveston, TX USA; 3 School of Biomedical Informatics, The University of Texas Health Science Center at Houston, Houston, TX USA

**Keywords:** Ion channels in the nervous system, Microarrays

## Abstract

Altered expression of GABA receptors (GABA_A_Rs) has been implicated in neurological and psychiatric disorders, but limited information about region-specific GABA_A_R subunit expression in healthy human brains, heteromeric assembly of major isoforms, and their collective organization across healthy individuals, are major roadblocks to understanding their role in non-physiological states. Here, by using microarray and RNA-Seq datasets—from single cell nuclei to global brain expression—from the Allen Institute, we find that transcriptional expression of GABA_A_R subunits is anatomically organized according to their neurodevelopmental origin. The data show a combination of complementary and mutually-exclusive expression patterns that delineate major isoforms, and which is highly stereotypical across brains from control donors. We summarize the region-specific signature of GAB_A_R subunits per subject and its variability in a control population sample that can be used as a reference for remodeling changes during homeostatic rearrangements of GABA_A_R subunits after physiological, pharmacological or pathological challenges.

## Abstract (web summary)

Adolfo Sequeira et al. report an analysis of GABA receptor subunit expression from single-cell and global transcriptome data generated by the Allen Institute. They find that GABA receptor subunit expression is spatially organized according to neurodevelopmental origin and generate a reference from the control population for future studies.

## Introduction

Gamma-aminobutyric acid (GABA) is the main inhibitory neurotransmitter in the adult central nervous system (CNS). Its actions are carried by the opening of anionic channels called GABA_A_ receptors (GABA_A_Rs), which are also pharmacological targets for drugs like benzodiazepines, barbiturates, ethanol, and nootropics, among many others^1–4^. The kinetics, physiology, and pharmacology of pentameric GABA_A_Rs are largely determined by their subunit stoichiometry. With 19 genes coding for individual subunits [α(1-6), β(1-3), γ(1-3), ρ(1-3), δ, θ, ε and π], the possible number of heteropentameric combinations is quite large. However, studies in animal models have shown that only few combinations are present in native tissue, with some of them more abundant than others in a region-specific pattern^1–3,5^. Because of the fundamental role of GABA_A_Rs in the control of neural excitability, changes in expression of these receptors have been implicated in neurological and psychiatric disorders characterized by alterations of the excitation to inhibition balance (e.g., epilepsy, autism spectrum disorders, schizophrenia, and major depression)^6–15^. Most gene expression studies have compared individual GABA_A_R subunits between diseased and control brains leading to a great understanding of severe Mendelian disorders^7,16^; however, figuring out the role of individual subunits in multifactorial and complex brain diseases has been more challenging, and studying only a few subunits may not be sufficient. The heteropentameric nature of GABA_A_Rs suggests that pathological changes in one or more subunits may remodel the stoichiometry of GABA_A_Rs and consequently their functional and pharmacological properties. So far, the most available way to measure the expression of all subunits is by microarray or RNA sequencing (RNA-Seq) technologies. Here again, large gaps of information of the healthy brain still complicate the analysis of GABA_A_Rs in complex brain disorders. Among the major roadblocks to understanding alterations of GABA_A_Rs in diseased states are scattered information of region-specific GABA_A_R subunit expression in healthy human brains^17,18^, unknown heteromeric assembly of predominant regional isoforms^1,19^, and lack of information about typical heterogeneity in the collective organization of GABA_A_Rs across healthy individuals^20^. In an effort to address these three issues, we performed four levels of analysis using publicly available data. The first analysis used microarray datasets from the Allen Institute to delineate major relationships between GABA_A_R subunits across 111 brain structures in six healthy human brains^21^. This analysis featured a low number of subjects but was extensive and comprehensive in its anatomical coverage. For the second analysis, we used RNA-Seq data from the Aging, Dementia and Traumatic Brain Injury (ADTBI) study, which has a high number of subjects (*n* = 56) but is limited to four regions: the hippocampus, the temporal and parietal cortices, and the white matter of the forebrain. For the third and fourth analyses, we used the RNA-seq dataset from the Allen Institute cell type study (13,348 single-cell nuclei from the medial temporal gyrus (MTG) of two subjects) to determine the coexpression of subunits according to their major classes (excitatory vs inhibitory) and their cell types (24 excitatory neurons and 45 inhibitory neurons). Together, these analyses provide complementary views of the relationships between GABA_A_R subunits in control human brains across different layers of complexity. We found that patterns of expression of GABA_A_R subunits are topographically organized according to their ontogenic origin and show high consistency in brain regions characterized by recurrent, or repetitive, cytoarchitecture at the regional and substructural levels. In contrast, subcortical regions composing the limbic and hypothalamic axis systems, which are often affected in neurological and psychiatric disorders, show high differential enrichment of specific GABA_A_R subunits. Moreover, some GABA_A_R subunits consistently show complementary or mutually exclusive expression patterns across the brain that delineate major heteropentameric assemblies. We also present a method to summarize the expression of the 19 genes in one metric that quantifies the organizational layout of GABA_A_R subunits within brain areas per subject, allowing for the calculation of the variability in the collective expression of brain structures in population studies. The organizational layout of GABA_A_R subunits in physiological conditions should help in determining their regional changes and remodeling in pathological conditions, and guide pharmacological strategies that target specific brain regions and functions by modulating GABA_A_Rs highly enriched in regions of interest.

## Results

### Global and region-specific brain expression

**Fig. 1:**
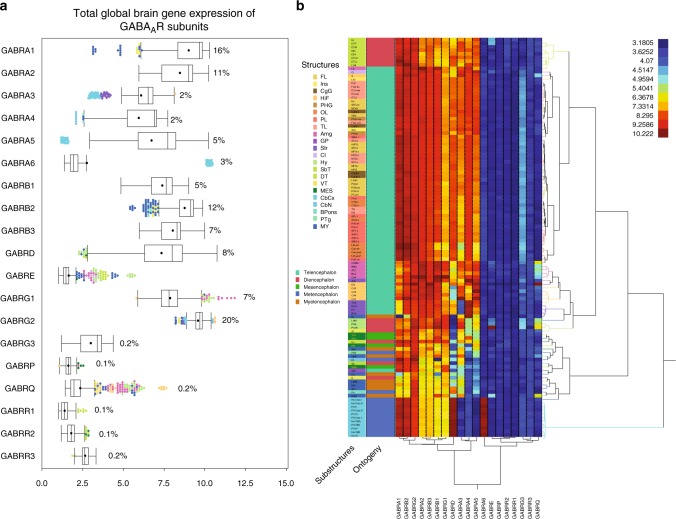
Global and region-specific gene expression of GABA_A_ receptor (GABA_A_R) subunits in the human brain. **a** Box plots of microarray gene expression of public available data from the Allen Brain Atlas (111 substructures per 6 subjects). The median is represented by the line within the box, and the first and third quartiles are represented by the ends of the box. The whiskers extend from each end of the box to the first or third quartile ±1.5 (interquartile range). Structures out of the whiskers are outliers and color coded according to the inset. The percentage shown is the proportional contribution (%) of each GABA_A_R subunit to the total expression in the brain. Total expression is the sum of the non-Log expression of the 19 genes across the 111 substructures in the brain. The general mean ± SD is 5.4 ± 3.2. **b** Two-way unsupervised Ward’s hierarchical clustering shows separation of major brain regions based on the Log2 gene expression; GABA_A_Rs subunits also clustered together according to their level of expression in each region (e.g., *GABRA1*, *GABRB2*, and *GABRG2* are in the same cluster). Labels for brain substructures and ontogenic origin are colored as per the insets. FL frontal lobe, Ins insula, CgG cingulate gyrus, HiF hippocampal formation, PHG parahippocampal gyrus, OL occipital lobe, PL parietal lobe, TL temporal lobe, Amg amygdala, GP globus pallidus, Str striatum, Cl claustrum, Hy hypothalamus, SbT subthalamus, DT dorsal thalamus, VT ventral thalamus, MES mesencephalon, CbCx cerebellar cortex, CbN cerebellar nuclei, Bpons basal part of the pons, PTg pontine tegmentum, MY myelencephalon. For substructure abbreviations, please see Supplementary Table 2

For analysis of gene expression of GABA_A_R subunits in the whole brain, we selected the most representative probe for each gene (Supplementary Data 1) from the Allen Brain Atlas Microarray Study according to the flowchart in Supplementary Fig. 1. The brain was divided into major regions, structures, and substructures following the Allen Brain Atlas nomenclature (Supplementary Data 2). This analysis showed three major gene expression profiles. Genes with background or noise levels of expression (Log_2_ < 4) across the brain (ρ1−3, π); genes with low global expression but high region-specific expression (α6, θ, γ3, ε); and genes with high expression all across the brain (Fig. 1a and Table 1). Among the high-expression genes, those for α1, β2 and γ2 contributed up to 48% of the global gene expression for GABA_A_Rs across the brain in the microarray dataset. Meanwhile, genes for α2, δ, β3, γ1, α5, and β1 contributed up to 43% of the total expression and genes for α3 and α4 up to 4%. As a group, these 11 genes contributed for ≈95% of total mRNA coding for GABA_A_Rs in the human brain (Fig. 1a). The second group with high region-specific expression subunits is of particular interest for pharmacological targeting of function/regions of the brain as these GABA_A_Rs are expressed at particularly high levels in discrete areas of the brain (Table 1).

**Table 1:** Anatomical enrichment of the GABA_A_Rs subunits across the human brain

Subunit	Major region	Structure	Structure abbreviation	Substructure abbreviation	Expression (Log2)	Fold enrichment
*GABRA1*	Cerebral cortex	Occipital lobe	OL	Cun-str	10.22	2.30
*GABRA2*	Cerebral nuclei	Amygdala	Amg	LA	10.24	3.38
*GABRA3*	Cerebral cortex	Hippocampal formation	HiF	CA1	7.98	3.68
*GABRA4*	Cerebral nuclei	Striatum	Str	Acb	7.70	3.34
*GABRA5*	Cerebral cortex	Hippocampal formation	HiF	CA2	10.09	10.21
*GABRA6*	Cerebellar cortex	Cerebellar cortex	CbCx	PV-V	10.45	204.81
*GABRB1*	Cerebral nuclei	Amygdala	Amg	CeA	8.85	2.73
*GABRB2*	Cerebral cortex	Occipital lobe	OL	Cun-str	9.76	1.98
*GABRB3*	Cerebral cortex	Hippocampal formation	HiF	DG	9.89	3.59
*GABRD*	Cerebellar cortex	Cerebellar cortex	CbCx	PV-VIIB	10.34	7.94
*GABRE*	Hypothalamus	Hypothalamus	Hy	PrOR	5.41	13.48
*GABRG1*	Cerebral nuclei	Amygdala	Amg	CeA	11.27	10.47
*GABRG2*	Cerebral cortex	Hippocampal formation	HiF	DG	10.63	2.05
*GABRG3*	Hypothalamus	Hypothalamus	Hy	PrOR	4.26	2.37
*GABRP*	Mesencephalon	Mesencephalon	MES	RN	2.27	1.56
*GABRQ*	Cerebral cortex	Hippocampal formation	HiF	DG	7.34	31.41
*GABRR1*	Thalamus	Dorsal thalamus	DT	ILr	2.45	2.05
*GABRR2*	Mesencephalon	Mesencephalon	MES	RN	2.60	1.72
*GABRR3*	Myelencephalon	Myelencephalon	MY	IO	3.20	1.44

The expression (Log2) represents the average expression per brain region and the fold enrichment the increase in the expression compared to the global average

*CA1* CA1 field, *Acb* nucleus accumbens, *CA2* CA2 field, *PV-V* V, paravermis, *CeA* central nucleus, *Cun-str* cuneus, striate, *DG* dentate gyrus, *GABA*_*A*_*R* GABA_A_ receptor, *PV-VIIB* VIIB, paravermis, *PrOR* preoptic region, *RN* red nucleus, *ILr* rostral group of intralaminar nuclei, *IO* inferior olivary complex

The expression pattern of GABA_A_R subunits was relatively homogenous within the cerebral and cerebellar cortices (Fig. 1b). A more heterogeneous gene expression profile was observed within subnuclei of the hippocampal formation, amygdala, basal ganglia, pons, and myelencephalon (MY). More specifically, the hippocampal formation, amygdala, and hypothalamus contained substructures with the more region-specific expression of particular subunits (Table 1). For instance, the highest expression for the γ2, β3, and θ subunits was found in the dentate gyrus of the hippocampus. Similarly, the central nucleus of the amygdala had the largest expression of β1 and γ1, and the preoptic region of the hypothalamus expressed the most ε and γ3.

The cerebellar cortex had the highest expression of α6 and δ genes, and the nucleus accumbens in the striatum had the highest expression of the α4 subunit (Fig. 1b and Table 1). The highest enrichment of subunits within these substructures was for α6 in the fifth lobule of the paravermis (PV-V), with a 205-fold of overexpression compared to the average across the brain, followed by dentate gyrus (θ enriched by 31-fold), preoptic region (ε, 13-fold), and central amygdala (γ1, 10-fold). These regions with particular expression of GABA_A_R subunits might be pharmacologically targeted to modulate GABAergic neurotransmission in a region/function-specific way. Unsupervised hierarchical clustering (UHC) dendrograms, based on patterns of gene expression of the individual GABA_A_R, revealed that most major structures clustered according to their anatomical localization. Untransformed (non-Log2), transformed (Log2), and proportional contribution of each subunit (average percentage that each subunit represented over the sum of all 19 GABA_A_Rs subunits; Supplementary Fig. 2) per substructure data produced dendrograms that showed clear separation of four major clusters: the cerebral cortex and cerebral nuclei, the dorsal thalamus, and the cerebellar cortex, which originate from the telencephalon, diencephalon, and metencephalon, respectively (Fig. 1b and Supplementary Fig. 3)^22,23^.

**Fig. 2:**
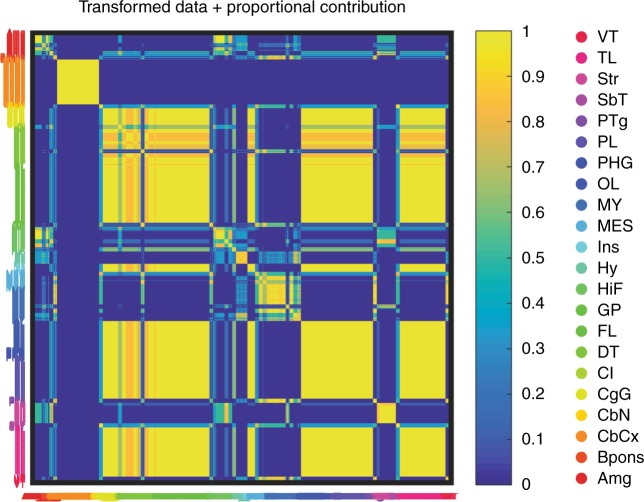
Quantum clustering secondary clustering of GABA_A_ receptor (GABA_A_R) subunits across the brain. Colors denote the fraction of co-occurrence of two structures together across different scale parameters, transformed data (Log2 of gene expression), and proportional contribution (%) of each GABA_A_R subunit to the total mRNA pool in each substructure. Labels for brain substructures are colored as per the inset shown at the farthest right. FL frontal lobe, Ins insula, CgG cingulate gyrus, HiF hippocampal formation, PHG parahippocampal gyrus, OL occipital lobe, PL parietal lobe, TL temporal lobe, Amg amygdala, GP globus pallidus, Str striatum, Cl claustrum, Hy hypothalamus, SbT subthalamus, DT dorsal thalamus, VT ventral thalamus, MES mesencephalon, CbCx cerebellar cortex, CbN cerebellar nuclei, Bpons basal part of the pons, PTg pontine tegmentum, MY myelencephalon

The amygdala, striatum, and hippocampus, which are also of telencephalic origin, were clustered adjacently to each other and next to the cortical areas. The subiculum, which is in the transition zone between the hippocampus and the cerebral cortex and is categorized as part of the hippocampal formation, clustered with the lateral amygdala and claustrum. The results of the secondary clustering from both the quantum clustering (QC) and spectral co-clustering (SCC) methods display similar patterns to the hierarchical clustering (Fig. 2 and Supplementary Fig. 4). Specifically, the cerebellar cortex structures show the most robust clustering and are tightly clustered together mainly because of the high levels of expression of *GABRA6*. The next robust clustering was of the cerebral cortex followed by the clustering between hippocampus, amygdala, and striatum pairs. In addition, gene expression for ρ1, ρ2, and ρ3 were also robust across clustering methods and parameters and consistently clustered with the MY and ventral thalamus structures. Finally, gene expression for ε and θ were also co-clustered together.

### Correlation of proportional contributions of GABA_A_R subunits

**Fig. 3:**
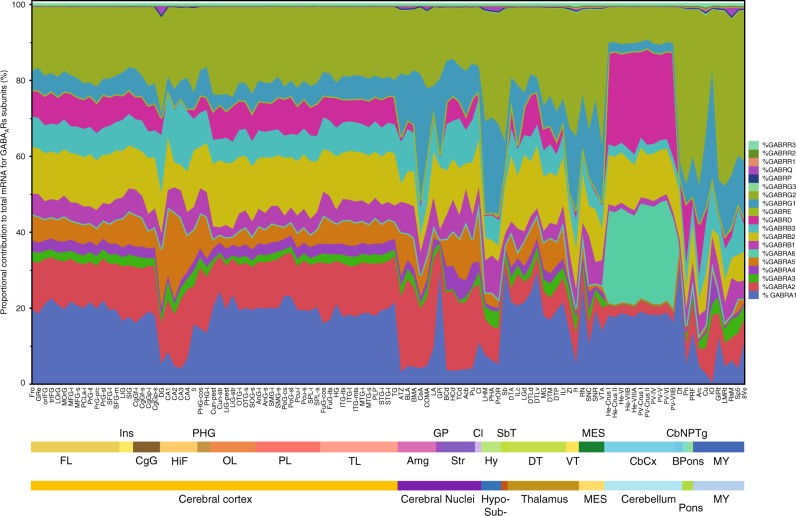
Region-specific proportional contribution of GABA_A_ receptors (GABA_A_Rs) across the brain. Each colored band represents the average percentage of gene expression of each subunit to the total pool of GABA_A_R subunit in the brain (*n* = 6 subjects). *y* axis displays the contribution as percentage. *x* axis displays brain structures in rostro-caudal order, and color coded, according to the Allen Brain Atlas. Proportional contribution per substructure is the single percent value for each gene. FL frontal lobe, Ins insula, CgG cingulate gyrus, HiF hippocampal formation, PHG parahippocampal gyrus, OL occipital lobe, PL parietal lobe, TL temporal lobe, Amg amygdala, GP globus pallidus, Str striatum, Cl claustrum, Hy hypothalamus, SbT subthalamus, DT dorsal thalamus, VT ventral thalamus, MES mesencephalon, CbCx cerebellar cortex, CbN cerebellar nuclei, Bpons basal part of the pons, PTg pontine tegmentum, MY myelencephalon. For substructure abbreviations, please see Supplementary Table 2

**Fig. 4:**
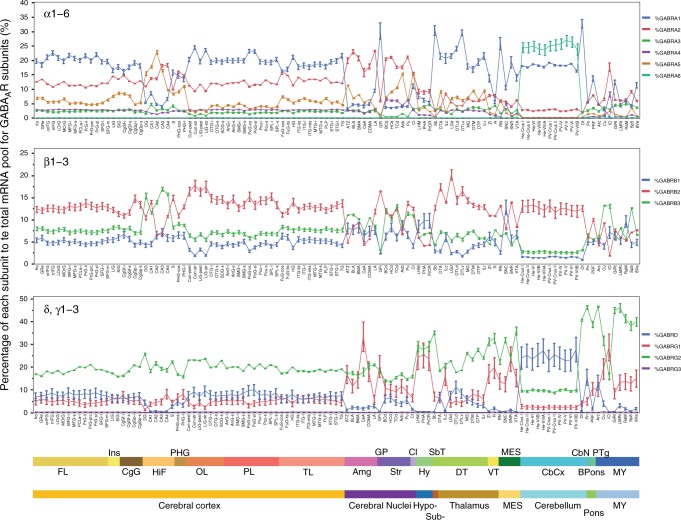
Global proportional contribution of gene expression by major subunit families. Mean ± SD of proportional contribution (%) of each subunit to the total pool of GABA_A_ receptor subunits (*n* = 6 subjects). *y* axis displays proportional contribution as percentage. *x* axis displays brain structures in rostro-caudal order, and color coded, according to the Allen Brain Atlas. FL frontal lobe, Ins insula, CgG cingulate gyrus; HiF, hippocampal formation; PHG, parahippocampal gyrus; OL, occipital lobe; PL, Parietal lobe; TL, temporal lobe, Amg amygdala, GP globus pallidus, Str striatum, Cl claustrum, Hy hypothalamus, SbT subthalamus, DT dorsal thalamus, VT ventral thalamus, MES mesencephalon, CbCx cerebellar cortex, CbN cerebellar nuclei, Bpons basal part of the pons, PTg pontine tegmentum, MY myelencephalon. For substructure abbreviations, please see Supplementary Table 2

Most pentameric GABA_A_Rs are made up by the combination of at least three different subunits from different families in a 2:2:1 pattern (2α plus 2β and one of either: γ, δ, ε, or π); therefore, it is expected that the amount of proteins for subunits assembled in major GABA_A_R isoforms, and ubiquitously expressed in the brain, should be linearly correlated at the global brain level. Whether the same is true for mRNA is not known, though a high overlap between protein and mRNA expression levels has been observed for GABA_A_Rs^24–27^. To investigate the patterns of collinearity between subunits across the brain, we first calculated the average percentage each subunit represented over the sum of all 19 GABA_A_R subunits expressed in each substructure for the 111 substructures analyzed (Fig. 3). This proportional contribution represents the available pool of GABA_A_R subunits mRNA in a particular brain region, structure, or substructure, and normalizes distinct levels of expression between different brain areas (e.g., cerebral cortex vs cerebral nuclei). Notably, many GABA_A_R subunits showed opposite patterns of expression. Opposite patterns between α1 vs α2, β1 vs β2 and γ1 vs γ2 are evident in Fig. 4 as mirror images of gene expression across the brain. Similar opposite patterns of expression were observed across the different brain areas of the ADTBI study (Supplementary Fig. 5) and across the 76 different cortical cell types from the cell-type Allen study (Supplementary Fig. 6).

**Fig. 5:**
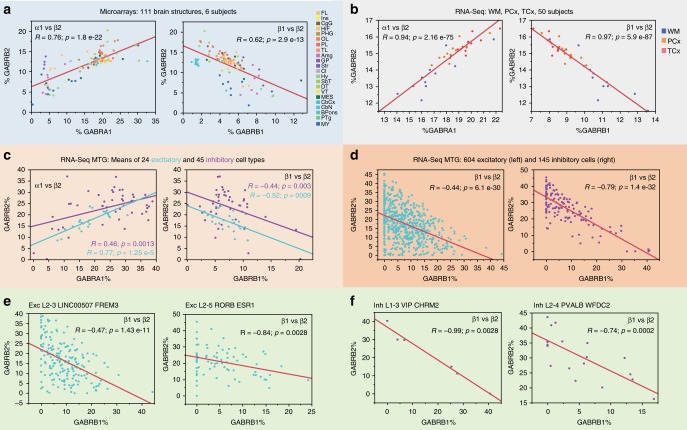
Correlation analysis between the most expressed GABA_A_ receptor subunits in microarrays and RNA-Seq. **a** Pearson correlations between the average of proportional contribution (%) of three genes that are significantly correlated across the brain using microarray datasets (*n* = 111 substructures; *r* and *p* values are shown as insets). **b** Same correlations using RNA-Seq datasets from the Aging, Dementia, and TBI study of the Allen Institute. *N* = 143 samples, 3 brain regions, from 52 subjects. **c** Linear correlations across distinct cell types using the mean of gene expression of all nuclei measured in each cell type (*N* = 12,560 nuclei), every dot is the mean of gene expression for each cell type. **d**–**f** Negative correlations using only nuclei in which the proportions of the three different components able to assemble in a pentameric receptor, Σ*α*, Σ*β*, and Σ*χ*, are close to a 2:2:1 proportions for all excitatory cells (**d**) or within distinct cell types (**e**, **f**)

To better understand the relationships between GABA_A_R subunits, the 11 genes that contributed for ≈95% of total expression of GABA_A_Rs were chosen for correlation analyses. Strong positive and negative correlations between specific GABA_A_R subunits across the brain were clearly observed (Fig. 5a). The strongest positive correlation was between α1 and β2 (*r* = 0.76; *p* = 1.8e−22; *n* = 111 substructures, Supplementary Data 3), followed by between α2 and β3 and between α5 and β3, suggesting that these subunits are co-regulated and there is an increased probability that they may be present as pairs in the same receptors. Interestingly, strong negative correlations were observed between α3 and δ (*r* = −0.71; *p* = 2.3e−18) and between β1 and δ, as well as between β1 and β2, which are members of the same family (a comprehensive list of *r* and *p* values can be found in Supplementary Data 3). The δ subunit, which can substitute for either of the γ subunits, was negatively correlated with γ1 across the brain.

Similar positive and negative correlations where observed when the temporal and parietal cortices and the white matter from the ADTBI were pooled together (Fig. 5b). Cross-correlation analysis of GABA_A_R subunits of the 3 brain regions (*n* = 143 samples from 50 subjects) showed strong positive correlations between subunits known to be assembled in native receptors (e.g., α1 and β2, *r* = 0.95; *p* = 2.2e−75; a comprehensive list of *r* and *p* values can be found in Supplementary Data 4). On the other hand, β1 and β2 subunits were negatively correlated (*r* = −0.97; *p* = 5.9e−88) as well as α1 with α2, β1, γ1; α2 with α4, β1, γ2; and γ1 with γ2. This indicated that the correlations between subunits were persistent at the global brain level and across a large number of subjects.

Because it might be possible that these correlations are not due to co-expression in the same cells but rather variability of cell types populations across brain areas, we analyzed RNA-Seq of the cell-type Allen Institute dataset. Single-cell nuclei data from this study had previously been classified in non-neuronal and neuronal cell classes^28^. Neuronal classes were classified in 24 glutamatergic and 45 GABAergic cell-type clusters based on their total gene expression (Supplementary Fig. 6). An initial PCA of the 19 GABA_A_R subunits for all 13,348 nuclei showed 2 highly overlapped populations that corresponded to excitatory and inhibitory neurons (Supplementary Fig. 7A). Further separation of excitatory vs inhibitory neurons was obtained when the mean of each gene per each of the 76 cell clusters was used for PCA (Supplementary Fig. 7B), indicating that the large variability in nuclei expression within each cell types hampers the collective analysis of GABA_A_Rs gene expression. Moreover, gene expression of GABA_A_R subunits in all cell-type clusters was extremely variable, with a large number of nuclei showing no expression of whole families of GABA_A_R subunits (Supplementary Fig. 8A).

To address this, we first analyzed the data within the context of the known pentameric structure of GABA_A_Rs and the complementary relationships found in this study (Fig. 4, Supplementary Figs. 5 and 6). Thus we determined the frequency distribution of three parameters: the sum of all *GABRAx* subunits (Σα = α1... + ...α6), the sum of all *GABRBx* + *GABRQ* subunits (Σβ = β1 + β2 + β3 + θ), and the sum of all subunits able to ensemble in the odd position (Σχ = γ1 + γ2 + γ3 + δ + ε + π), that are present in each nucleus. Remarkably, the means for Σα, Σβ, and Σχ were close to 40%, 40%, and 20%, respectively (Supplementary Fig. 8A), which correspond to the 2:2:1 proportions expected for pentameric GABA_A_Rs. Further analysis showed that the larger the number of nuclei sampled within each cell type the more Σα, Σβ, Σχ converge to 40%, 40%, and 20%, respectively (Supplementary Fig. 8B), indicating that most variability within each cell cluster is due to low number of nuclei sampling. Based on this information, for correlation analysis by cell-type clusters we only used datasets from nuclei in which the range of proportional contribution for Σα and Σβ was 40 ± 5% and for Σχ was 20 ± 5%. Remarkably, positive and negative correlations were consistently found when GABA_A_R subunits were correlated across different cell-type populations (Fig. 5c, Supplementary Data 5) or within each cell type (Fig. 5d–f and Supplementary Data 6). Interestingly, a strong negative correlation between *GABRB2* and *GABRB3* was evident at the single-cell level that was not observed when using whole-brain regions (complete list of the correlations can be found in Supplementary Data 5, 6, and 7). Table 2 shows the summary of all subunits pairs that were correlated in two or more of the analyses and are most likely shared (positive correlations), or mutually excluded from (negative correlations), the same receptor.

**Table 2:** Pairwise correlation analysis of subunit gene expression across levels, from single cell to global brain expression

Positive correlations					Negative correlations				
Gene	Gene	Subunits	From datasets	Cell class (C,D)	Gene	Gene	Subunits		Cell class (C,D)
*GABRA1*	*GABRB2*	α1−β2	A,B,C,D	Exc, 1 Inh	*GABRA1*	*GABRA2*	α1, α2	B,C,D	9 Exc, 2 Inh
*GABRA1*	*GABRD*	α1−δ	B,D	1 Exc	*GABRA1*	*GABRA3*	α1, α3	B,D	1 Exc
*GABRA2*	*GABRA4*	**α2**−**α4**	**A,C**	**Exc, Inh**	*GABRA1*	*GABRA4*	α1, α4	C,D	4 Exc, 2 Inh
*GABRA2*	*GABRB1*	α2−β1	A,B		*GABRA1*	*GABRA5*	α1, α5	D	2 Exc, 1 Inh
*GABRA2*	*GABRB3*	α2−β3	A,D	1 Inh	*GABRA1*	*GABRB1*	α1, β1	A,B,C,D	1 Exc, 1 Inh
*GABRA3*	*GABRB1*	α3−β1	A,B,C,D	1 Exc	*GABRA1*	*GABRB3*	α1, β3	A,C	
*GABRA4*	*GABRB2*	α4−β2	B,D	1 Exc	*GABRA1*	*GABRG1*	α1, γ1	A,B,C	
*GABRA4*	*GABRD*	α4−δ	B,C	Exc	*GABRA2*	*GABRA4*	**α2**, **α4**	**B,D**	**1 Exc**
*GABRB1*	*GABRB3*	**β1−β3**	**A,C**	**Inh**	*GABRA2*	*GABRB2*	α2, β2	B,C,D	1 Exc, 1 Inh
*GABRB1*	*GABRG1*	β1−γ1	A,B		*GABRA2*	*GABRG2*	α2, γ2	B,D	1 Inh
					*GABRA3*	*GABRD*	α3, δ	A,B	
					*GABRA5*	*GABRB2*	α5, β2	B,C	Inh
					*GABRA5*	*GABRG2*	α5, γ2	B,C	
					*GABRA5*	*GABRG2*	α5, δ	B,C	
					*GABRB1*	*GABRB2*	β1, β2	A,B,C,D	2 Exc, 4 Inh
					*GABRB1*	*GABRB3*	**β1, β3**	**D**	**2 Exc**
					*GABRB1*	*GABRAD*	β1, δ	A,B,C,D	1 Exc
					*GABRB2*	*GABRB3*	β2, β3	D	11 Exc, 3 Inh
					*GABRB2*	*GABRG1*	β2, γ1	A,B,C,D	1 Exc
					*GABRB3*	*GABRG2*	β3, γ2	C,D	1 Inh
					*GABRG1*	*GABRG2*	γ1, γ2	B,D	4 Exc
					*GABRG1*	*GABRD*	γ1, δ	A,B	
					*GABRG2*	*GABRD*	γ2, δ	A,D	4 Exc, 3 Inh

Dataset A: Allen Atlas Microarray study, Dataset B: Aging, Dementia and Traumatic Brain Injury study, Datasets C and D: Allen Institute Cell-type study across excitatory and inhibitory cells and within each cell-type cluster, respectively. Cell class was determined from datasets C and D. Subunits in bold indicate pairs with positive and negative correlations across different analyses

Information in Table 2 supports the presence of human receptors with stoichiometries previously identified in animal models and listed by Olsen and Sieghart in 2008 (e.g., α1β2γ2, α4β2γ2, α4β2δ). It also supports the presence of receptors that are likely but have not been confirmed (e.g., α1β2δ and α2β1γ1). Additional evidence toward the existence of functional α2β1γ1 receptors in humans is provided by the observation that astrocytes only express genes for these three subunits (Supplementary Figure 6), and astrocytes are known to express functional GABA_A_Rs^29^. Perhaps the most interesting result is the identification of negative correlations between subunits. The presence of negative correlations across substructures with different cytoarchitectural make-up and distinct ontogenic origin, across different cell types, and across cells of the same type are strong indicators of potential mutual exclusion. Therefore, our analysis argues against the presence of receptors containing pairs of subunits listed in Table 2, particularly those that were observed as mutually exclusive in all four distinct analyses. For example, the existence of receptors with α1β1γ1 and α2β2γ1 stoichiometry or β1 and β2 in the same receptor is unlikely. Notice that β1−β3 had positive correlations in inhibitory cells but negative in two excitatory cell types; similarly, α2−α4 also showed positive and negative correlations, suggesting a cell-dependent modulation of these subunits.

### Variability of collective organization across individuals

**Fig. 6:**
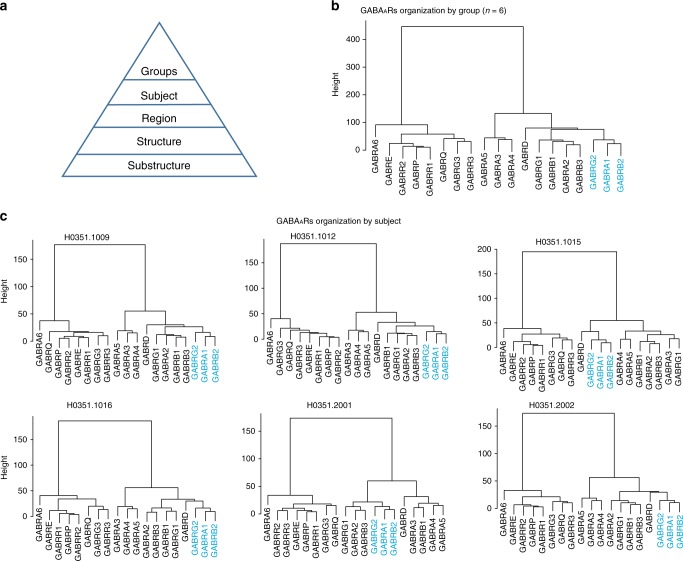
Global organizational layout of GABA_A_ receptor (GABA_A_R) subunits. **a** Euclidean distance comparisons can be used to identify the organizational layout of GABA_A_Rs at different levels. **b** Dendrograms showing the whole-brain association between subunits at the group level (*n* = 6 subjects) using transformed (Log2) microarray data, notice the similarity with the subunits clustering in Fig. 1b. **c** Individual dendrograms showing the global association of subunits within each subject

Potential variation between individuals may complicate our ability to generate a reliable reference map of GABA_A_R subunit expression in the human brain. Therefore, we used the Euclidean distances (*d*) between expression levels of each subunit to evaluate the extent of global organizational variation between individuals. First, inter-individual variability in the microarray cohort of the Allen Brain Atlas (*n* = 6 subjects) was quantified by calculating *d*_i_ (individual) and *d*_c_ (consensus) between the expression levels of each subunit and correlating these results (Supplementary Fig. 9). The correlation coefficient (*R*) between the individuals and the consensus reference was high (*R*(*d*_i_, *d*_c_) = 0.998 ± 0.0007; mean ± SEM)). Hierarchical clustering of the consensus data assists in visualizing associations between the different subunits (Fig. 6b). Two major conclusions can be made from the dendrogram. First, all subunits clustered on the right are highly expressed throughout the brain, and all subunits clustered on the left are expressed at noise levels except α6, ε, and θ, which have high expression only in specific brain regions (See also Fig. 1). Second, gene expression for α1, β2, and γ2 were clustered together and α2, β1, β3, and γ1 were closely associated. Hierarchical clustering of each subject (Fig. 6c) shows a high degree of variability in the relative positioning of each subunit and cluster but no variability in the clustering of the subunits themselves, particularly the α1, β2, and γ2 clusters.

**Fig. 7:**
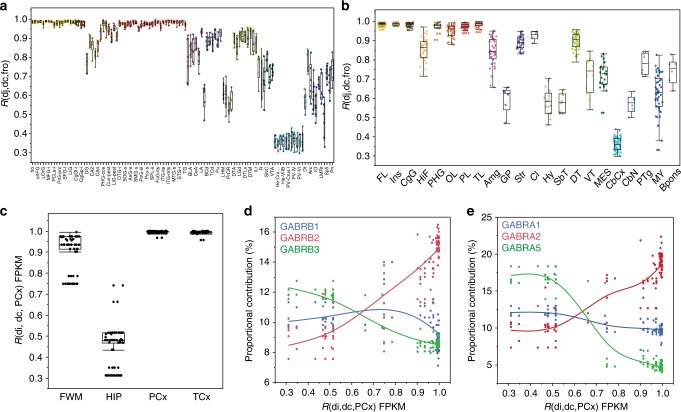
Correlation coefficients of Euclidean distances as a metric of variability. **a**, **b** The Euclidean distances between subunits per structure per subject can be correlated against a standard (frontal operculum, fro) to show variability in subunit expression between substructures (**a**) or structures (**b**). Correlation coefficients (*R*) closer to one indicate higher similarity in expression patterns between the structure and the standard. Each dot is a single subject containing the information of the 19 GABA_A_ receptor subunits. The box plots elements are defined as in Fig. 1. Brain regions are ordered in anteroposterior axis and color coded as in Fig. 1. **c** Euclidean distances from gene expression (fragments per kilobase of transcripts per million mapped read) and proportional contribution data from the ADTBI study were correlated against the parietal cortex. Again, coefficients closer to one indicate higher similarity between the structure and the parietal cortex. **d**, **e** Plots of proportional contribution against the correlation coefficients of the parietal cortex. Proportional contribution clearly shows opposite patterns between subunits. FL frontal lobe, Ins insula, CgG cingulate gyrus, HiF hippocampal formation, PHG parahippocampal gyrus, OL occipital lobe, PL parietal lobe, TL temporal lobe, Amg amygdala, GP globus pallidus, Str striatum, Cl claustrum, Hy hypothalamus, SbT subthalamus, DT dorsal thalamus, VT ventral thalamus, MES mesencephalon, CbCx cerebellar cortex, CbN cerebellar nuclei, Bpons basal part of the pons, PTg pontine tegmentum, MY myelencephalon. For substructure abbreviations, please see Supplementary Table 2

Similar methods can be employed to determine the variability between individuals across brain areas at different anatomical levels (Fig. 6a). To compare the brain regions at the level of substructures the frontal operculum (Fro), which is the most frontal structure in the Allen Atlas, was chosen to construct a reference (consensus) against which all other 110 substructures were compared (*d*_c,Fro_). Each substructure from each subject was then measured against this reference (Fig. 7a). Minimal variability between individuals, as shown by high correlation values, was observed in frontal cortical areas, when using transformed (*R*(*d*_i_, *d*_c,Fro_) = 0.985 ± 0.005) or proportional contribution data (*R*(*d*_i_, *d*_c,Fro_) = 0.979 ± 0.007; Supplementary Fig. 10). Most cortical areas were similar to the reference and showed minimal inter-individual variability, except the occipital cortex, which was significantly different from the reference, and also was more variable between subjects (analysis of variance (ANOVA) results and *p* values are in Supplementary Data 8). We also compared brain areas at the structural level using the same approach (Fig. 7b; ANOVA results and *p* values are in Supplementary Data 8).

Clear differences in inter-structure heterogeneity and inter-individual variability are easily observed. For example, when compared to the reference, hippocampal regions were more heterogeneous and more variable between individuals (*R*(*d*_i_, *d*_c,Fro_) = 0.85 ± 0.008) than the cerebral cortex but less than amygdalar nuclei or myelencephalic areas. The most distinct region from the neocortex was the cerebellar cortex (*R*(*d*_i_, *d*_c,Fro_) = 0.36 ± 0.006), and the most heterogeneous and variable region between individuals was the MY. The remarkable minimal variation of the organizational layout of GABA_A_Rs between temporal and parietal cortex across control individuals, and its dissimilarity to hippocampus and white matter in terms of expression and variability across individuals, was confirmed using the parietal cortex as a reference (*d*_c,PCx_) in the RNA-Seq dataset of the ADTBI study (Fig. 7c). Hippocampus samples were the most variable across individuals (ANOVA results and *p* values are in Supplementary Data 9). The variability was explained by differences in the coordinated expression of GABA_A_Rs subunits. The closer *R*(*d*_*i*_, *d*_c,PCx_) is to 1, the larger the expression of the subunits α1 and β2 and the lower the expression of α2, α5, β1, and β3 (Fig. 7d, e).

## Discussion

Our multiple clustering analyses indicate that gene co-expression patterns of GABA_A_R subunits are region-specific and cluster according to embryonic structures. This suggests that co-expression patterns are determined early during development and relatively stable during adulthood within structures characterized by recurrent cytoarchitecture, like the cerebral and cerebellar cortices^22^. On the contrary, subcortical structures are more heterogeneous indicating greater diversity in inhibitory signaling processing, which is congruent with a more complex neuroanatomical cytoarchitecture. This heterogeneity also emphasizes the differential modulatory effects that the diverse subcortical nuclei have on the activity of distinct cortical regions^30,31^. A detailed characterization of the GABA_A_Rs patterns using functional rather than anatomical organizational principles will surely provide additional information in future studies.

Our observation of GABA_A_Rs expression patterns in brain regions clustered based on their embryonic origins is in agreement with previous studies which have shown that a large number of genes have a region-specific expression closely following their embryonic origin^32,33^. It is also congruent with the early role of GABA_A_Rs in the modulation of cell division, migration, and differentiation^34–36^ and later with their participation in the generation of electrical and synaptic activity in developing neurons^37,38^. Expression patterns of GABA_A_Rs change during development^18,39^; however, the related patterns of GABA_A_R subunits expression of substructures within the amygdala, hippocampus, and striatum indicate that control mechanisms of phenotypic expression are partially shared among most telencephalic structures and partly differentiated into nuclei-specific patterns. A shared pattern may arise from a common embryonic structure, like the telencephalic interneurons that migrate from the ganglionic eminence to cortical areas^40–44^, and further nuclei-specific differentiation is likely due to distinct developmental gene expression programs^45–47^ and activity-dependent cell specification^48,49^. Although to our knowledge the molecular determinants of GABA_A_R subunits expression in humans are still not known, elegant work by Mulligan et al.^50^ identified several gene candidates in mice models that may participate in the coordinated expression of multiple subunits. Future research about human homologs of those candidates and their role in the developmental specificity of GABA_A_Rs is needed.

There was a large overlap between the expression of GABA_A_Rs in humans with accumulated evidence from animal models; however, there were also several important differences. Similar to previous observations in animal models and meta-analytical studies, the most abundant GABA_A_R mRNA subunits in the cerebral cortex were α1, α2, β2, and γ2. Positive correlations between these subunits indicate that heteromeric receptors composed of α1β2γ2 subunits are the most abundant across the cerebral cortex^1,2,50–53^. Interestingly, α1 was negatively correlated with β1 and γ1 across the brain, suggesting a mutually exclusive relationship. Similarly, negative correlations suggest mutual exclusion between α1 and α2 and between β1 and β2. The mutual exclusion between α1 and α2 subunits is at odds with co-immunoprecipitation data from animal models. Although the majority of GABA_A_Rs receptors have one type of α subunit^54^, there are evidence of receptors containing both α1 and α2 subunits^54–56^. Owing to limited human tissue availability, to our knowledge no similar data currently exists for humans; however, opposite expression of α1 and α2 in the different substructures of the hippocampus, thalamus, and substantia nigra are also clearly observed in recent immunohistochemical studies^17,57,58^. Although the presence of GABAARs with a1 -a2 stoichiometry in humans cannot be discarded, their presence might be limited; if they do occur it may be due to posttranslational mechanisms. In the cerebellar cortex, our correlation analysis strongly suggests the presence of native pentameric stoichiometries α6β2δ, α1β2δ, and α1β2γ2. Similarly, the presence of α5β3 pairs is expected in the hippocampus, and because α5 and δ are negatively correlated, the most probable combination of α5-GABA_A_Rs is α5β3γ2. Interestingly, the dentate gyrus has the highest gene expression of the θ subunit, an important difference with animal models. This subunit was highly correlated with α1, δ, and γ2, suggesting the presence of receptors with α1θγ2 or α1θδ stoichiometry. Alternatively, it is tempting to speculate that θ could replace one β2 or β3 subunit and make up receptors with α1β2/3θγ2 stoichiometry. With the exception of the dentate gyrus, the expression of θ subunit in humans shows a striking overlap with the adult rodent brain and is highly correlated with expression of α3 and ε (Fig. 1b)^59–61^. The amygdala was the second most dissimilar brain region compared to the rest of the brain and expressed high levels of α2, β1, γ1, and ε, suggesting the presence of α2β1θε and α2β1θγ1 receptors. The preoptic region of the hypothalamus was the third most dissimilar expressing high levels of ε and γ3 subunits. Interestingly, the three most dissimilar structures according to GABA_A_R expression: dentate gyrus, central amygdala, and preoptic region, are also sites of adult neurogenesis in animal models^62,63^, which has been proposed as a mechanism of action of tricyclic antidepressants and selective serotonin reuptake inhibitors in these brain regions^64^. Moreover, the hippocampus, amygdala, and hypothalamus form part of two of the most critical networks in the brain, the limbic system, and the hypothalamic–pituitary–adrenocortical axis, often involved in neurologic and psychiatric disorders^65–67^. The fold enrichment of unique subunits in these regions (Table 1) makes them particularly interesting pharmacological targets to regulate nuclei activity via modulation of GABAergic neurotransmission. However, it is important to recognize that GABA_A_Rs have a large range of pharmacological interactions and most GABAergic drugs have shared effects on different GABA_A_Rs (Supplementary Fig. 11). Moreover, many GABAergic drugs are subunit selective only in a very narrow nM range before affecting a broad range of GABA_A_Rs subtypes^19^. Thus the development of drugs with specificity on subunits differentially enriched requires further development. For example, drugs with selectivity over the θ subunits are limited despite the large abundance of θ on regions with strong neurogenesis, such as the dentate gyrus. A promising example of targeted pharmacology is the efficacy of inverse agonists of α5-containing GABA_A_Rs in the hippocampus that reduce memory impairment following alcohol consumption^68^.

Noise-level expression of transcripts for ρ1, ρ2, and ρ3 across the brain in microarray datasets is consistent with studies reporting a limited and cell-specific expression for these genes in animal models^25,69–72^. Transcripts for π were at noise levels in microarray datasets and extremely low, if any, in RNA-Seq of the hippocampus and the temporal lobe. Although transcripts for π had been found in hippocampus and temporal cortex of humans^73^, little information about its distribution in the CNS is available, despite being identified as a susceptibility gene in schizophrenia^74^.

How are particular assemblies of GABA_A_Rs preferred over the many possibilities? Our correlation analysis argues in favor of a co-regulation of GABA_A_R subunits that are clustered in the same chromosome for the most abundant receptors in the brain (*cis* effects^20^), with a tight control by cell-specific transcription factors as seen in the temporal silencing of α6 in cerebellar granular cells^75^ or intrinsic signals that ensure mutual segregation^76^. Additional diversity of GABA_A_Rs across the brain is provided by coordinate transcription between genes located in different chromosomes (*trans* effects) similar to what is observed in mice^50^. Strong *cis* coordinated expression of subunits across brain regions suggest that remodeling of GABA_A_Rs during disease may happen within restricted and coordinated patterns. Evidence of this has been seen in alcohol and cocaine exposure^20,77^, Angelman syndrome^78^, and Alzheimer’s disease^27^, in which alterations of several subunits that are expressed in single chromosome clusters are collectively modified.

Probably the highest roadblock in comparing physiological and non-physiological brain states is the lack of information about whether the expression of GABA_A_Rs is stereotypical or highly variable across control individuals. Our results using Pearson correlation of Euclidean distances indicate that, despite high variation on the level of gene expression of individual subunits across control subjects, the collective organization of GABA_A_Rs in each brain structure is highly stereotypical, providing a framework for future studies exploring remodeling during diseased states. The combination of Euclidean distances and the proportional contribution of GABA_A_R subunits allows the quantification of population (cohort) variability, while identifying the subunits driving the variability.

The use of raw Euclidean distances to correlate pairs of GABA_A_R subunits in each region, across individuals, has some limitations that are common to other mathematical distances of the Minkoski family; for example, the large-scale feature dominates the rest^79^. However, this is a starting point from where we attempt to minimize the transformation of the data as much as possible. Moreover, the degree of dissimilarity is given by the correlation coefficient between the Euclidean distances of consistent sets of 19 gene values across subjects, and the lower the correlation coefficient, the larger is the extent of collective differences in subunit expression. Future studies focusing in evaluating distinct metrics of similarity and dissimilarity and correlation strategies^80^ for subunits of GABA_A_Rs and other neurotransmitter receptors across individuals are highly encouraged.

In conclusion, we show that the patterns of expression of GABA_A_Rs subunits in the brain is highly stereotypical across healthy controls. This consistent pattern within structures is observed even in regions with the highest intra-individual and intra-structure variation, like the hippocampus, amygdala, and hypothalamus. These regions are the most dissimilar to the rest of the brain and also show the most region-specific expression of GABA_A_Rs, underlining the opportunity to target specific regions to modulate GABA neurotransmission for precise pharmacological treatments targeting specific neuropsychiatric conditions. Future studies that include neurodegenerative and psychiatric RNA-Seq datasets should be useful to explore homeostatic rearrangements of GABA_A_R subunits after physiological, pharmacological, or pathological challenges.

## Methods

### Microarray and RNA-Seq databases

Three datasets, derived from public domain resources as described below, were used for this study: normalized microarrays from the Allen Brain Atlas at the sample resolution level (http://human.brain-map.org), normalized RNA-Seq datasets from gray matter (parietal cortex, temporal cortex, and hippocampus) and white matter of the forebrain from the ADTBI study (http://aging.brain-map.org/download/index), and normalized single-cell nuclei RNA-Seq datasets from the cell-type Allen study (http://celltypes.brain-map.org/rnaseq). For the microarray datasets, the detailed demographic characteristics of the 6 subjects (5 males and 1 female between 24 and 57 years of age with no known neuropsychiatric or neuropathological history), as well as technical white papers about tissue acquisition, data processing, normalization, and quality control procedures can be found at: http://help.brain-map.org/display/humanbrain/Documentation. For the RNA-Seq datasets of the ADTBI study, the demographic characteristics of 56 healthy controls (35 males and 21 females between 78 and 99 years of age with no history of neurodegenerative or psychiatric disorders) are also available in the downloading site. For single-cell analysis, we used an RNA-Seq dataset from two subjects, H200.1030 (54 year old, male) and H200.1023 (43 year old, female), that provided 84.8% of all data in the study. In total, 12,560 of neurons NeuN(+) (94.1%) and non-neuronal 788 NeuN(−) (5.9%) nuclei, from the 6 different cortical layers from these two subjects, were used for the initial analyses (Supplementary Figs. 2, 6, 7, and 8), and a subset of this cohort was used for the correlation analysis in Fig. 5 and Table 2.

Institutional Review Board review and approval was obtained for collection of tissue and non-identifying case information at the tissue banks and repositories that provided tissue to the Allen Atlas Institute; tissue was collected after informed consent from decendent’s next-of-kin (http://help.brain-map.org/display/humanbrain/Documentation). A diagram of the analysis flow and its relevance in the context of the study can be found in supplementary material (Supplementary Fig. 1).

### Selection of microarray probes

The Allen Brain Atlas used 246 probes to measure the expression of 19 GABA_A_Rs genes (e.g., 49 probes to test *GABRA1*) using a custom design (by Beckman Coulter Genomics) Agilent 8 × 60K array that includes the 4 × 44K Agilent Whole Human Genome probe set supplemented with an additional 16,000 probes. To avoid redundant clustering due to the co-linearity between probes for the same gene, only one probe per gene was selected. For genes with only two probes, the probe with the highest expression was used. When more than two probes per gene were available, a principal components-based Exploratory Factor Analysis with no rotation in JMP 12Pro identified the most representative probe (Supplementary Data 1). Selected probes were also consistent across human subjects using the method described by Kirsch and Checnik^33^, wherein the expression correlation across regions of each probe was computed, then the correlation scores across all pairs of subjects were averaged, and the most correlative probe was chosen. Selected probes mapped to most of the splices variants for each gene in Ensembl genome browser (https://useast.ensembl.org/index.html), indicating no specific bias, and that our results are more representative of the collective isoforms expressed for each gene. This is strengthened by the observation that the similarities in the proportional contribution of each selected probe to the total pool of GABA_A_Rs in the temporal cortex was highly correlated to the RNA-Seq gene expression patterns in the temporal lobe of ADTBI study and the MTG of the cell-type Allen study (Supplementary Fig. 2), thus validating the selection of representative gene probes in the microarray study. For descriptive analysis, the brain was divided into major regions, structures, and substructures (Supplementary Data 2) using information from the Allen Brain Atlas. Only substructures that were measured across all 6 subjects, except the white matter in microarray datasets, due to negligible expression, were included in this study.

### Analysis of data

Age effects on the levels of gene expression were corrected by linear regression in JMP version 14 discovery from SAS (JMP 14) using age as a continuous variable in the microarray datasets or as an ordinal value in the aging categories available in the ADTBI dataset. Sex and ethnicity had no effects on the mRNA expression levels in microarray or RNA-Seq datasets. For global expression in microarray analyses, the mean (*M*) ± standard deviation (SD) of GABA_A_R subunit expression for each structure, per subject, was calculated as the average of each substructure measurement, including left and right substructures, reported in the Allen Brain Atlas, unless stated otherwise. The proportional contribution of the total expression of each GABA_A_R subunit (Fig. 1a) was defined as the percentage of untransformed (non-Log2) expression level of each subunit to the total microarray expression of all GABA_A_R subunits across the brain, all of which add to 100%; the percentage of total expression of GABA_A_R subunits in the brain is equivalent to the area of one colored band in Fig. 3. The proportional contribution of each GABA_A_R subunit per substructure, or cell type, is the percentage of expression level of each subunit gene to the total pool of subunit genes within each brain substructure/cell type^27,81^. For this, the sum of non-Log2 microarray data, or fragments per kilobase of transcripts per million mapped reads in RNA-Seq data, of all 19 genes per substructure/cell type was 100%.

### Clustering analyses

UHC was done using the Ward’s minimum variance method, where the distance between two clusters is the ANOVA sum of squares between the two clusters added up over all the variables. Ward’s method joins clusters to maximize the likelihood at each level of the hierarchy (JMP 14). We also used two additional clustering methods to assess the robustness of our clusters: QC^82^ and SCC^83^.

QC is based on physical intuition derived from quantum mechanics. It starts by constructing scale-space probability function from the data points and derives a potential function by viewing the latter as the lowest eigenstate of a Schrodinger equation. QC requires only one parameter, which determines the scale over which cluster structures are searched. Looking at clustering results at different scales enables the identification of robust clusters. Furthermore, it has been recently shown that QC has the advantage of an unbiased analysis by filtering out the weight information from the density function, while focusing on the shape of the data, thus allowing of detection of clusters of different densities^84^. Prior to applying QC, we preprocessed the data by taking only the top five principal components of the singular value decomposition (accounting for >80% of the variability of the data) and applying a whitening transformation. SCC, on the other hand, aims to identify biclusters of structures and GABA subunits. SCC requires the specification of the number of biclusters. In order to utilize both the QC scale parameter and the SCC cluster number parameters, we conducted a secondary clustering in which we varied these parameters over a large set of scales and cluster numbers and computed the percentage of times any two structures or GABA subunits in SCC co-appear in the same cluster.

### Euclidean distances analyses

Pearson product–moment was used for all the linear correlations. To estimate the population variation of GABA_A_R transcriptional organization between subjects, we used the coefficient of correlation (*R*) between the Euclidean distances (*d*_i_) of expression levels for the 19 subunits per each subject and the consensus Euclidean distances (*d*_c_) composed by all subjects. The Euclidean distances *d*_i_ and *d*_c_ were calculated as shown below:

1 $${d_{\mathrm{i}}({\mathrm{GABR}}x - {\mathrm{GABR}}y) = \sqrt {({\mathrm{GABR}}x - {\mathrm{GABR}}y)^2}}$$

2 $${d_{\mathrm{c}}({\mathbf{GABRx}} - {\mathbf{GABRy}})=}\\ {\sqrt {({\mathrm{GABR}}x_1 - {\mathrm{GABR}}y_1)^2 + ({\mathrm{GABR}}x_2 - {\mathrm{GABR}}y_2)^2 + \ldots + ({\mathrm{GABR}}x_n - {\mathrm{GABR}}y_n)^2}}$$

where *d*_i_ is the Euclidean distance of a pair of subunits in each individual; *x* and *y* indicate distinct GABA_A_R subunits; *d*_c_ is the consensus Euclidean distance; and *n* is the number of subjects in the group.

### Statistics and reproducibility

Data analyses and plotting were initially implemented in JMP 14 and then repeated in RStudio using R3.5.0 and the mosaic package for R Markdown with the same results. QC and SCC were implemented as a custom code of published algorithms using Matlab version 9.1.10. Unbiased QC and SCC provide similar results to UHC. For statistical comparison of brain substructures in Euclidean distance analysis, we used one-way ANOVA followed by multiple comparison with the consensus using Dunnet’s method; *p* < 0.05 was considered significant.

### Reporting summary

Further information on research design is available in the Nature Research Reporting Summary linked to this article.

## Supplementary information

Supplementary Data 1

Supplementary Data 2

Supplementary Data 3

Supplementary Data 4

Supplementary Data 5

Supplementary Data 6

Supplementary Data 7

Supplementary Data 8

Supplementary Data 9

Supplementary Data 10

Description of Additional Supplementary Files

Supplementary Material

Reporting Summary

## Data Availability

Microarray and RNA sequence data that support the findings of this study are available from the Allen Institute: http://human.brain-map.org, http://aging.brain-map.org/download/index, and http://celltypes.brain-map.org/rnaseq. Additional data used for the figures are provided in Supplementary Data 10.

## Code availability

All codes used in this work have been previously published and are publicly available. A pipeline of the analysis for fast implementation can be provided upon request.
