# Supplementary material for: Human brain transcriptome analysis finds region- and subject-specific expression signatures of GABAAR subunits
Source: Commun Biol. 2019 May 1;2:153. doi: 10.1038/s42003-019-0413-7 (PMC6494906; doi:10.1038/s42003-019-0413-7)
Supplement: Supplementary file 11 — Description of Additional Supplementary Files [file 42003_2019_413_MOESM11_ESM.pdf]

Description of Additional Supplementary Files

File Name: Supplementary Data 1
Description: Selected Probes by Exploratory Factor Analysis (Excel file)

File Name: Supplementary Data 2
Description: Major Brain Regions, structures and substructures abbreviations (Excel file)

File Name: Supplementary Data 3
Description: Pearson's correlation values for microarray datasets (Excel file)

File Name: Supplementary Data 4
Description: Pearson's correlation values for RNA sequencing datasets (Excel file)

File Name: Supplementary Data 5
Description: Pearson's correlation values for GABAergic vs Glutamatergic cells (Excel file)

File Name: Supplementary Data 6
Description: Pearson's correlation values by cell type cluster (Excel file)

File Name: Supplementary Data 7
Description: Correlations that cross hierarchies and cell types (Excel file)

File Name: Supplementary Data 8
Description: Statistical Analysis correlation of Euclidian distances Allen Microarray (Excel file)

File Name: Supplementary Data 9
Description: Statistical Analysis correlation of Euclidian distances ADTBI RNAseq study (Excel file)

File Name: Supplementary Data 10
Description: Dataset source for figures (Excel file)
