# Supplementary material for: Human brain transcriptome analysis finds region- and subject-specific expression signatures of GABAAR subunits
Source: Commun Biol. 2019 May 1;2:153. doi: 10.1038/s42003-019-0413-7 (PMC6494906; doi:10.1038/s42003-019-0413-7)
Supplement: Supplementary file 12 — Supplementary Material [file 42003_2019_413_MOESM12_ESM.pdf]

Human brain transcriptome analysis finds region- and subject-specific expression signatures of $GABA_AR$ subunits

Adolfo P. Sequeira, Kevin Shen, Assaf Gottlieb and Agenor Limon.

Supplementary Material

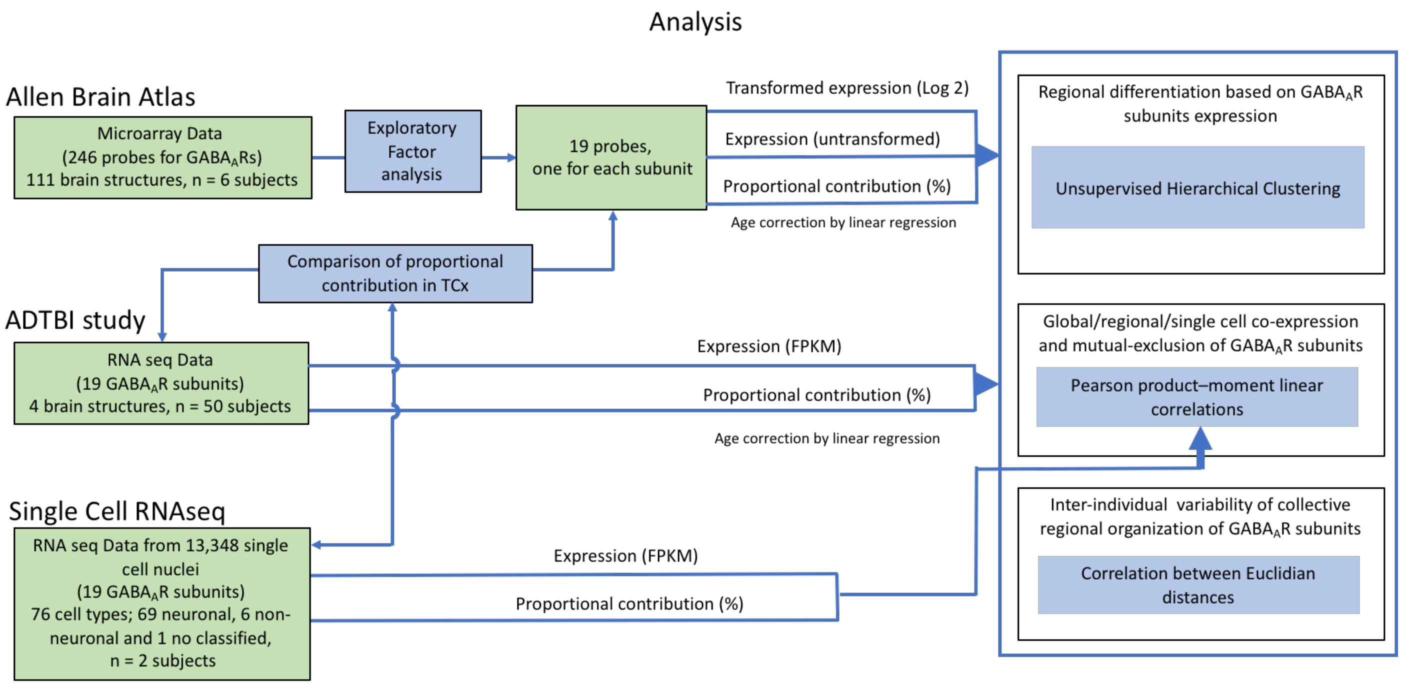

Supplementary Figure 1. Flowchart of the analysis. The diagram shows the cohorts, analysis and the context of the procedures used in this study.

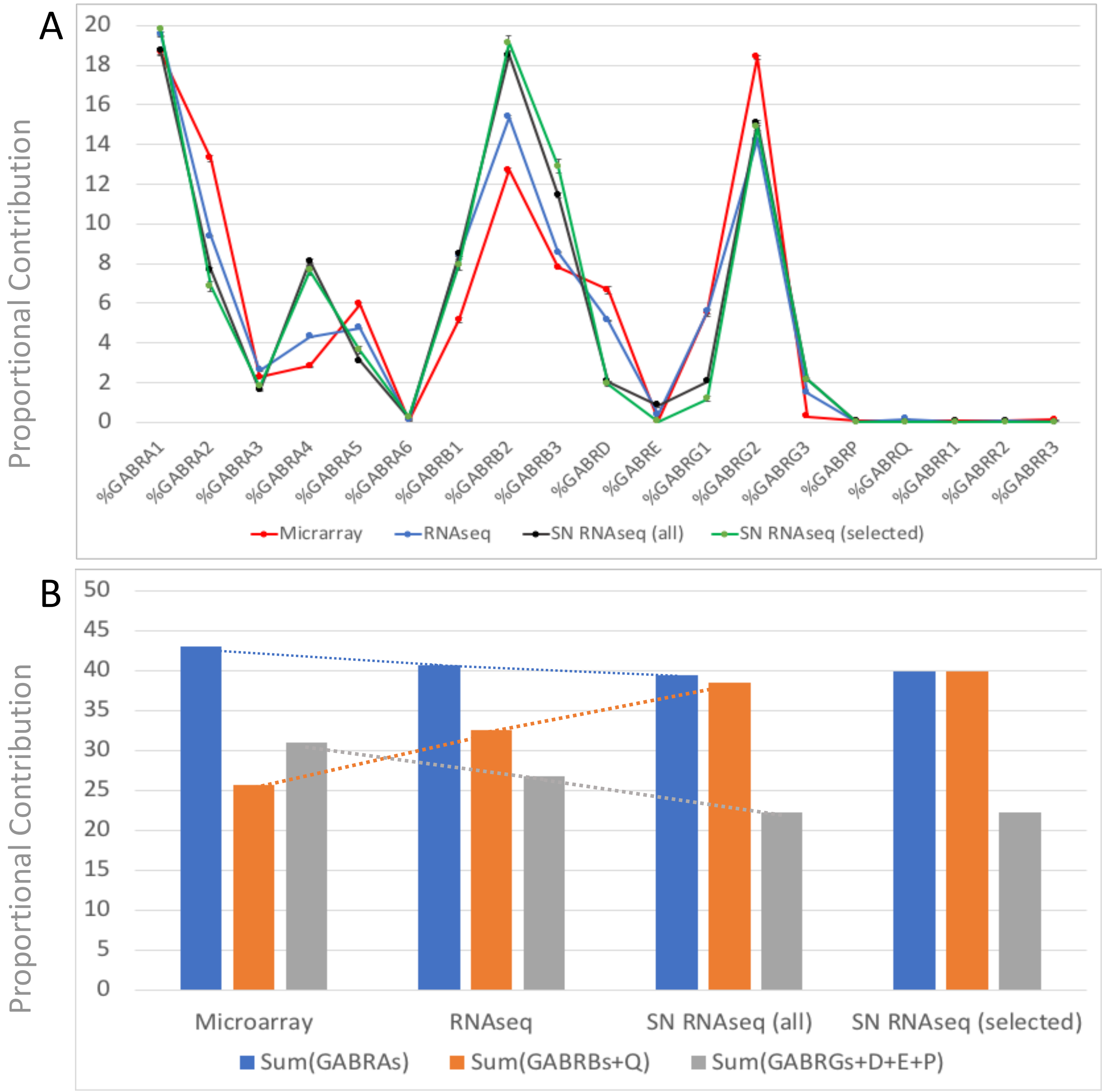

Supplementary Figure 2. Proportional contribution to the total pool of $GABA_AR$ subunits across different datasets involving the temporal cortex. A. Percentage of gene expression levels to the total of all $GABA_ARs$ subunits in the temporal cortex (Mean ± SEM) of public available data from the Allen brain atlas microarray study (red), the Aging, Dementia and Traumatic Brain Injury (ADTBI) study (blue), and the cell-type study of the Allen Institute (green and black). For the microarray analysis 72 independent measurements from 12 substructures and 6 control subjects were used. For the ADTBI RNA-Seq analysis the data was obtained from 50 subjects with non-dementia diagnosis and in which measurements of the temporal cortex were available. For the cell-type study two analysis were done: one that includes all 13,348 nuclei from 1 male and 1 female, and other with 757 selected nuclei, from the same subjects. The 757 nuclei selected have a 40 ± 5 % on their proportional contribution for the sum of all *GARBRAs* subunits, 40 ± 5 % for the sum of all *GABRBs* plus *GABRQ*, and 20 ± 5 % for the sum of all subunits that ensemble in the odd position. B. Percentage of the three different families of subunits according to their ensemble in a pentameric receptor for the three datasets used. Notice that as the resolution of gene expression and number of sampling increases the proportion of these parameters converge to a 2:2:1 relationship.

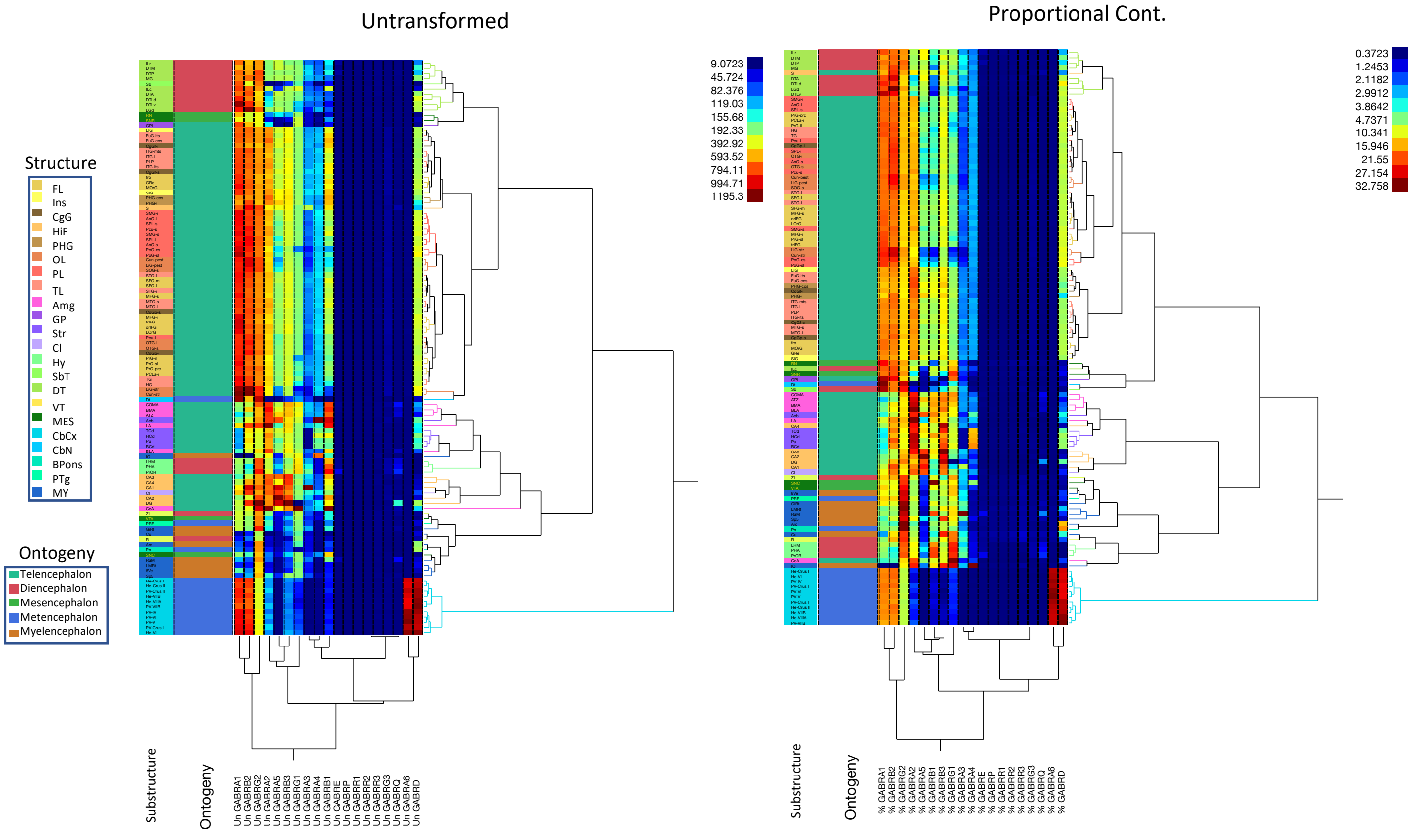

Supplementary Figure 3. Hierarchical clustering of GABA$_A$R subunits across the brain. Two ways unsupervised Ward's hierarchical clustering shows similar degrees of separation of major regions based on the format of the data used: averaged values per substructure (n = 6 subjects) for non-Log2 (left) and proportional contribution (right). Labels for brain substructures are colored as per the insert shown at the farthest left. Substructure labels are colored by structure and ontogenic origin. FL, frontal lobe; Ins, insula; CgG, Cingulate gyrus; HiF, hippocampal formation; PHG, parahippocampal gyrus; OL, occipital lobe; PL, Parietal lobe; TL, temporal lobe; Amg, amygdala; GP, globus pallidus; Str, striatum; Cl, claustrum; Hy, hypothalamus; SbT, subthalamus; DT, dorsal thalamus; VT, ventral thalamus; MES, mesencephalon; CbCx, cerebellar cortex; CbN, cerebellar nuclei; Bpons, basal part of the pons; PTg, pontine tegmentum; MY, myelencephalon. For substructures abbreviations please see Supplementary Table 2.

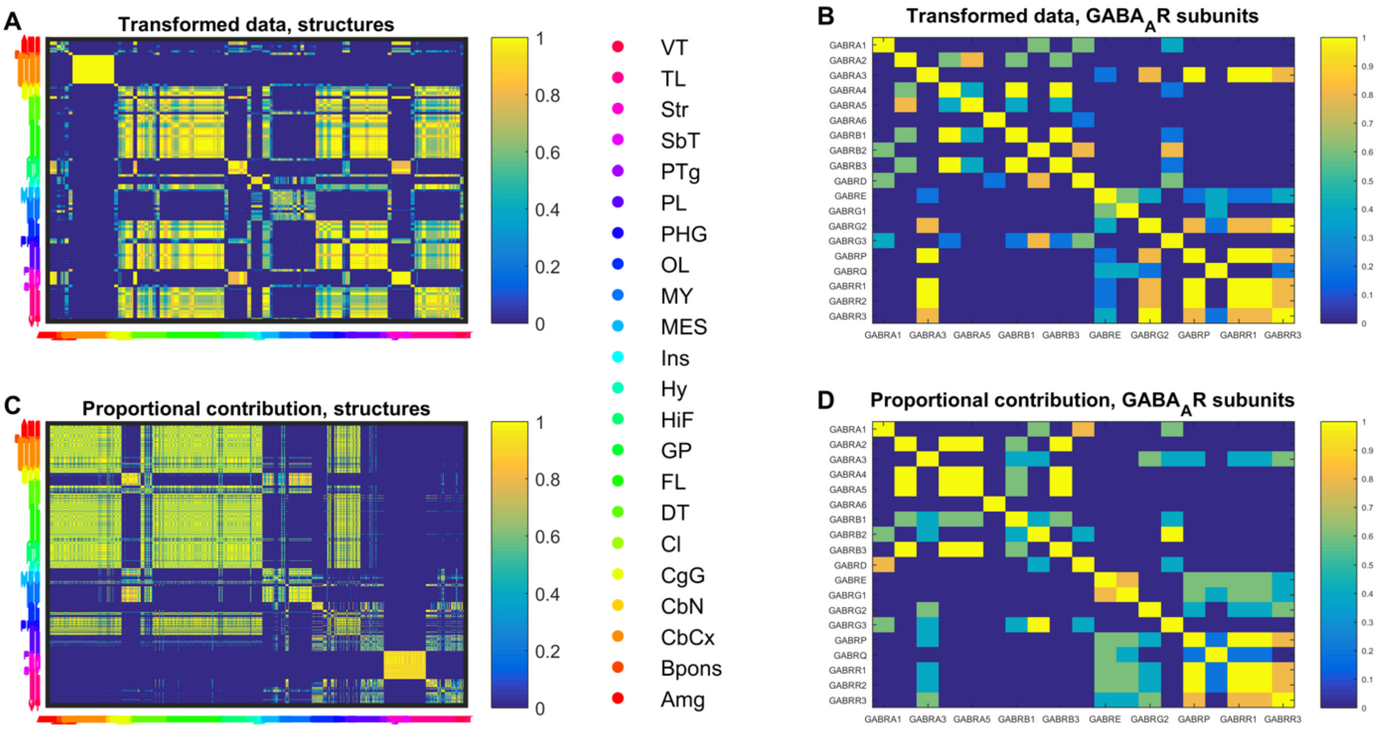

Supplementary Figure 4. Spectral co-clustering of structures and GABA$_A$R subunits across the brain. Displayed are the structures using the log-transformed data (A) and proportional contribution (C) and the GABA$_A$R subunits using the log-transformed data (B) and proportional contribution (D). FL, frontal lobe; Ins, insula; CgG, Cingulate gyrus; HiF, hippocampal formation; PHG, parahippocampal gyrus; OL, occipital lobe; PL, Parietal lobe; TL, temporal lobe; Amg, amigdala; GP, globus pallidus; Str, striatum; Cl, claustrum; Hy, hypothalamus; SbT, subthalamus; DT, dorsal thalamus; VT, ventral thalamus; MES, mesencephalon; CbCx, cerebellar cortex; CbN, cerebellar nuclei; Bpons, basal part of the pons; PTg, pontine tegmentum; MY, myelencephalon.

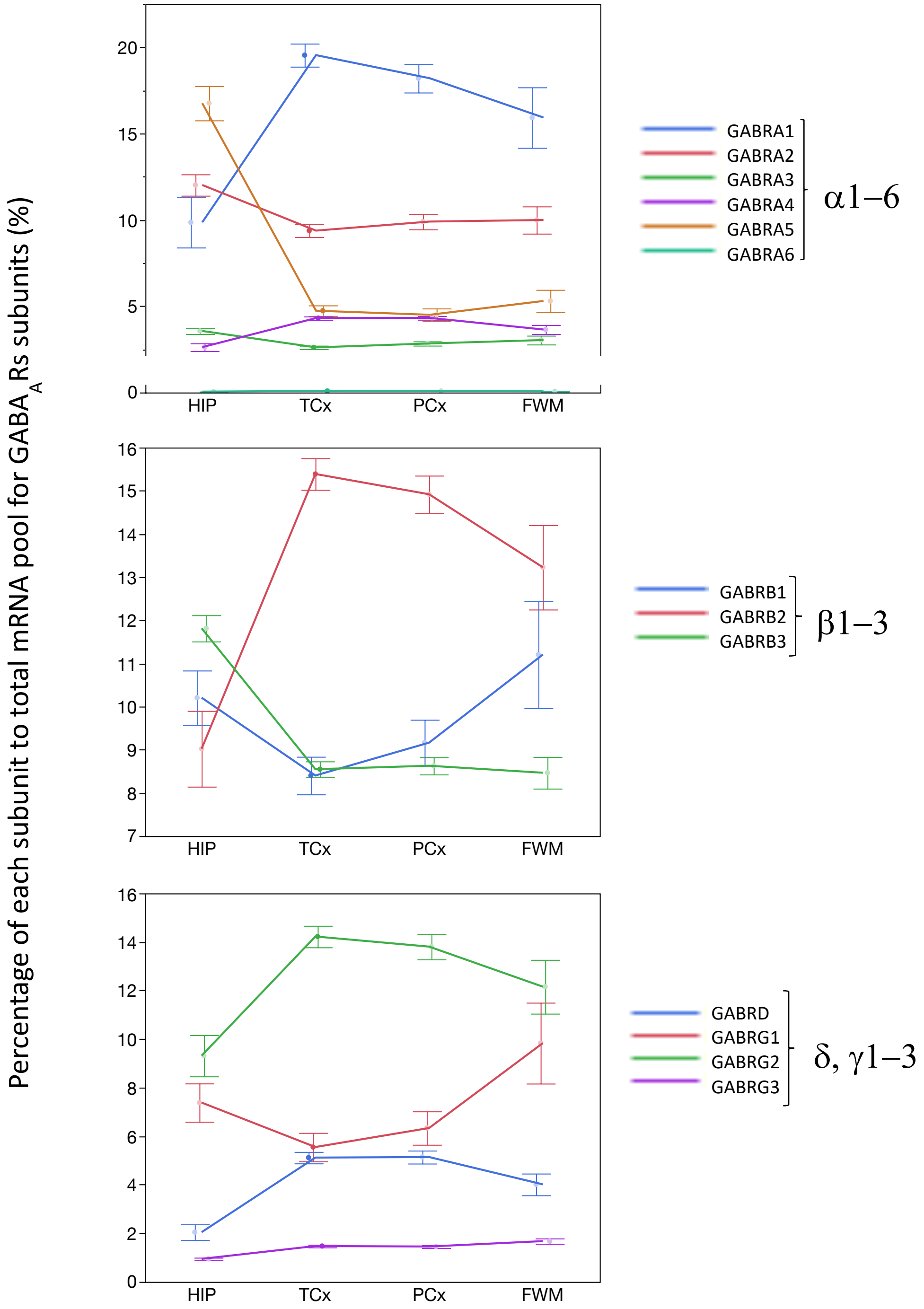

Supplementary Figure 5. Proportional contribution of gene expression by major subunit families. Mean ± SD of percentage of each subunit to the total pool of GABA$_A$R subunits (n = 56 subjects). The y-axis displays expression as percentage. The X-axis displays the hippocampus (HC), parietal cortex (PCx), temporal cortex (TCx) and white matter of the forebrain (WH). RNAseq data obtained from the Aging, Dementia, and TBI study of the Allen Institute.

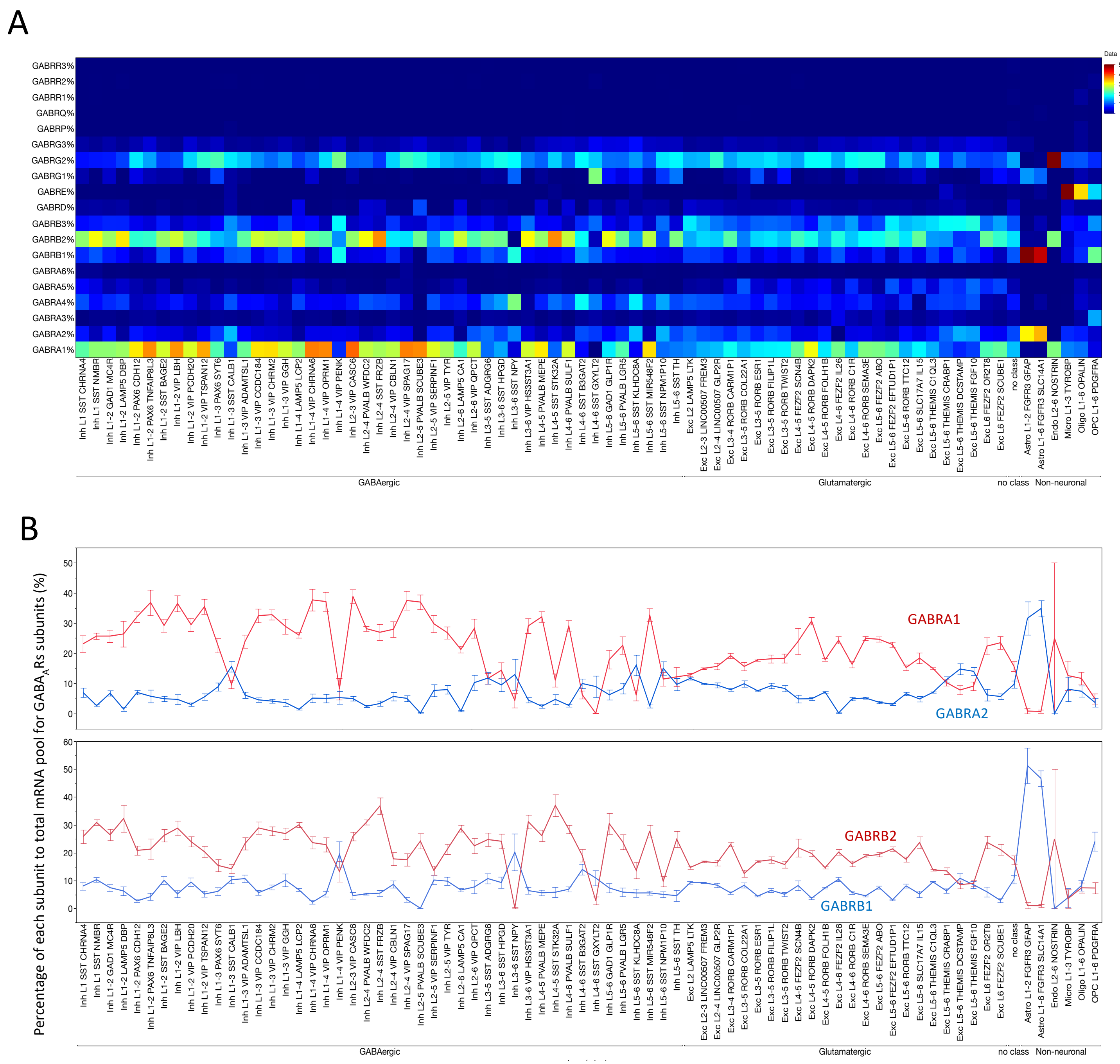

Supplementary Figure 6. Cell-type gene expression of $GABA_A$R subunits in the medial temporal gyrus. A. Heatmap of expression showing the mean of gene expression for all 76 cell types of the cell-type Allen Study (N = 13,348 nuclei). B. Mean ± SEM of proportional contribution (%) of four subunits to the total pool of $GABA_A$R subunits in each nucleus (N = 13,348 nuclei).

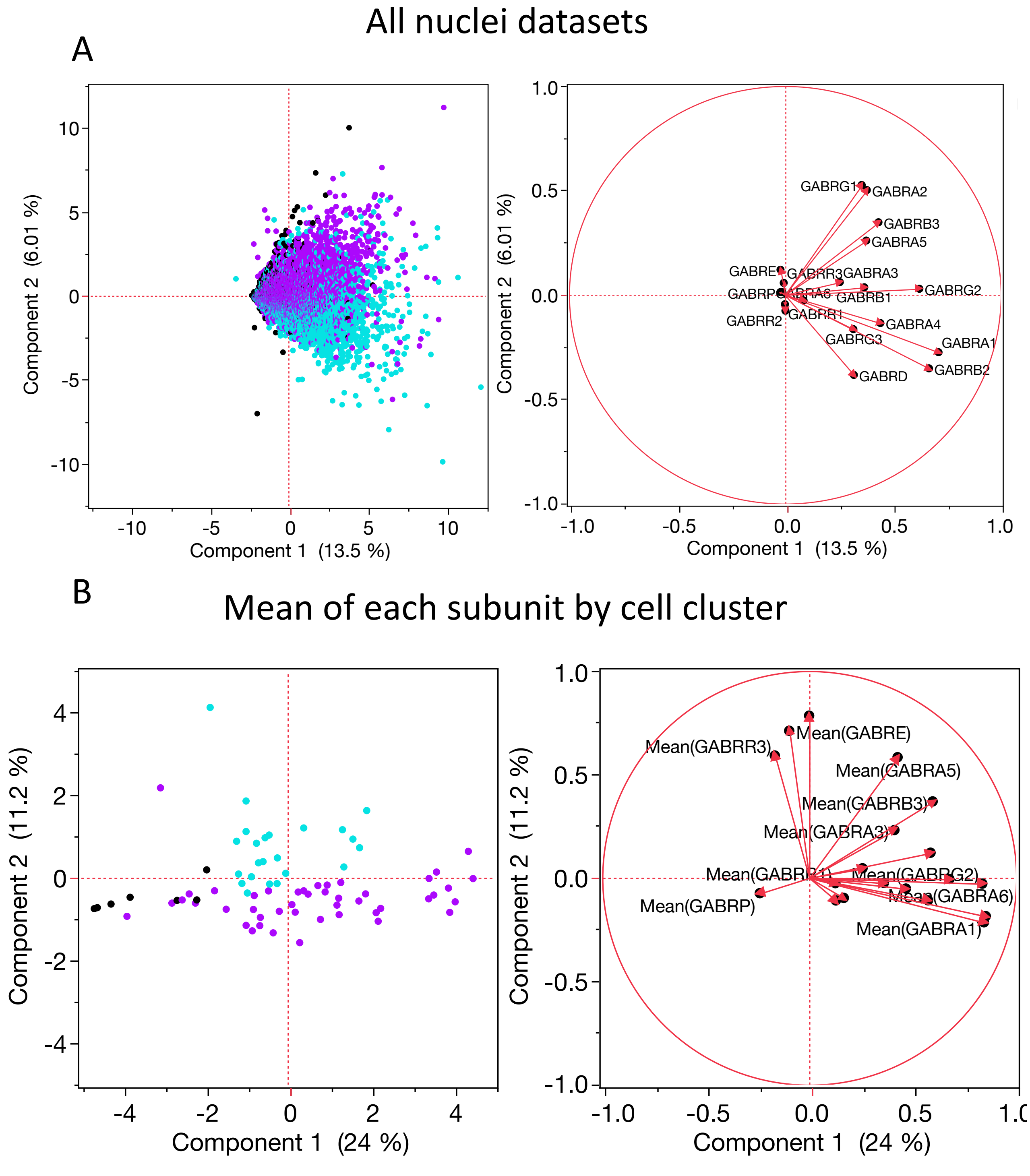

Supplementary Figure 7. Principal components analysis (PCA) of RNA-Seq in the cell type Allen Institute dataset. A) Scores and loading plots for the components 1 and 2, using the FPKM for all 19 $GABA_AR$ subunit genes in 13,348 nuclei isolated from the medial temporal gyrus (8,831 nuclei from 24 excitatory cell types (turquoise), 3,555 nuclei from 45 GABAergic cell types (magenta), 191 nuclei from a non-classified cell type (black) and 771 from no- neuronal cells (black). B) Same analysis as in A using the mean of gene expression for each subunit in each of the 76 different cell types. A list of the different cell types can be found in Supplementary Data 7.

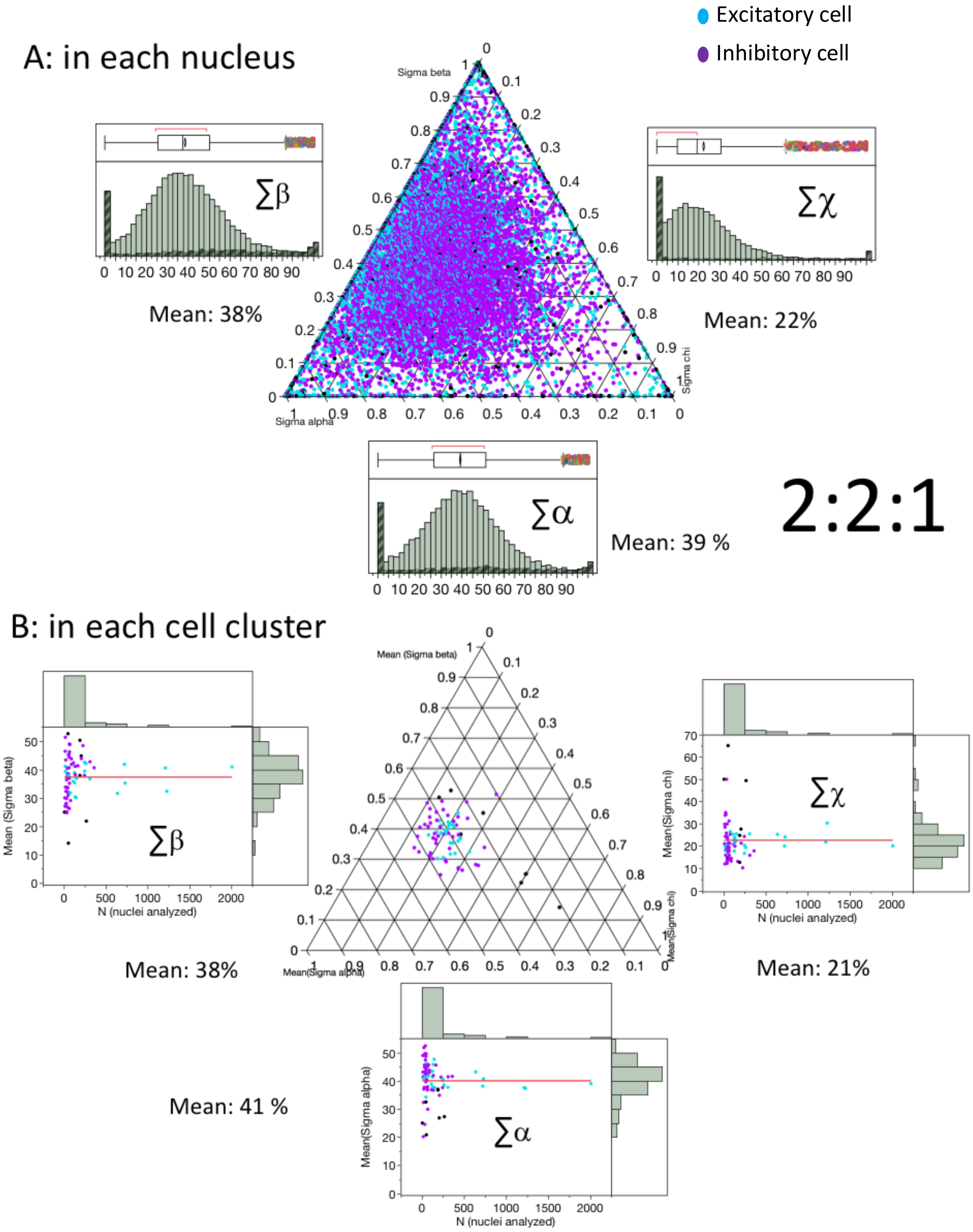

Supplementary Fig 8. Proportional contribution of the subunits according to their possible arrangement in a pentameric receptor. A) Ternary plot at the center of the panel indicates the proportional contribution of the three different components able to assemble in a pentameric receptor, $\Sigma\alpha = \alpha1..+..\alpha6$, $\Sigma\beta = \beta1+\beta2+\beta3+\theta$, and $\Sigma\chi = \gamma1+\gamma2+\gamma3+\delta+\varepsilon+\pi$, for each nucleus isolated from the medial temporal gyrus (N= 8,831 nuclei from 24 excitatory cell types (turquoise), 3,555 nuclei from 45 GABAergic cell types (magenta), 191 nuclei from a non-classified cell type (black) and 771 from non-neuronal cells (black). Histograms at the side of each axis indicate the distribution frequency of each parameter. Shadowed columns indicate the proportion of nuclei with no expression of whole members of single subunits families. Notice that regardless of the large variability the means for the three components show a 2:2:1 relationship. B) Ternary plot for the mean of the proportional contribution for each cell type (N = 76 cell types). Scatter plot of the mean of the three components vs the number of nuclei analyzed per cell type, notice that as the sampling increases the values converge towards a 2:2:1 relationship. Histograms show the distribution of values for x and y axis, and the red line is the fit to the mean for the y axis.

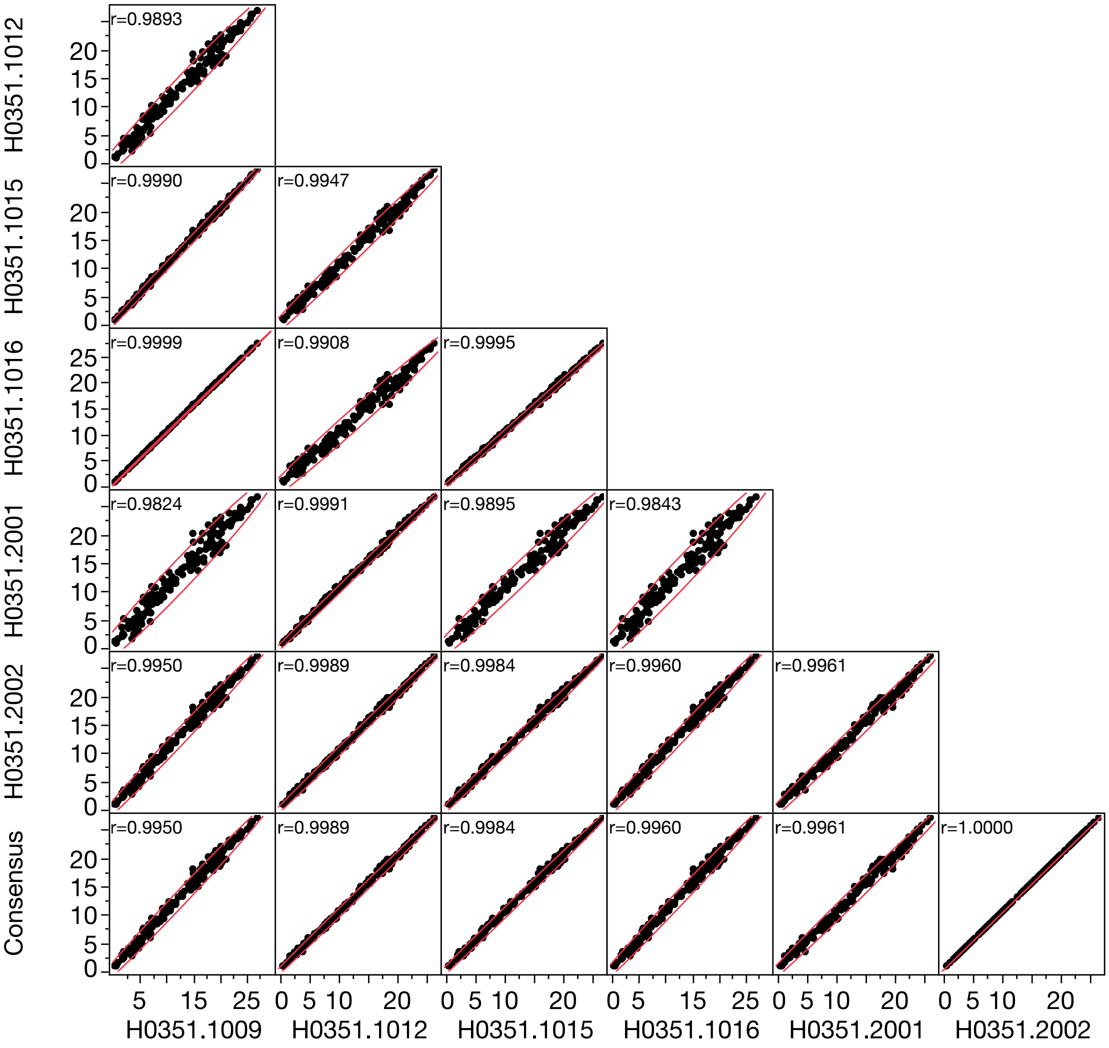

Supplementary Figure 9. Individual variability in subunit expression. Left, Pearson correlations across sets of $GABA_AR$ subunits Euclidean distances show a very high correlation ($r > 0.98$) between individuals and with the consensus.

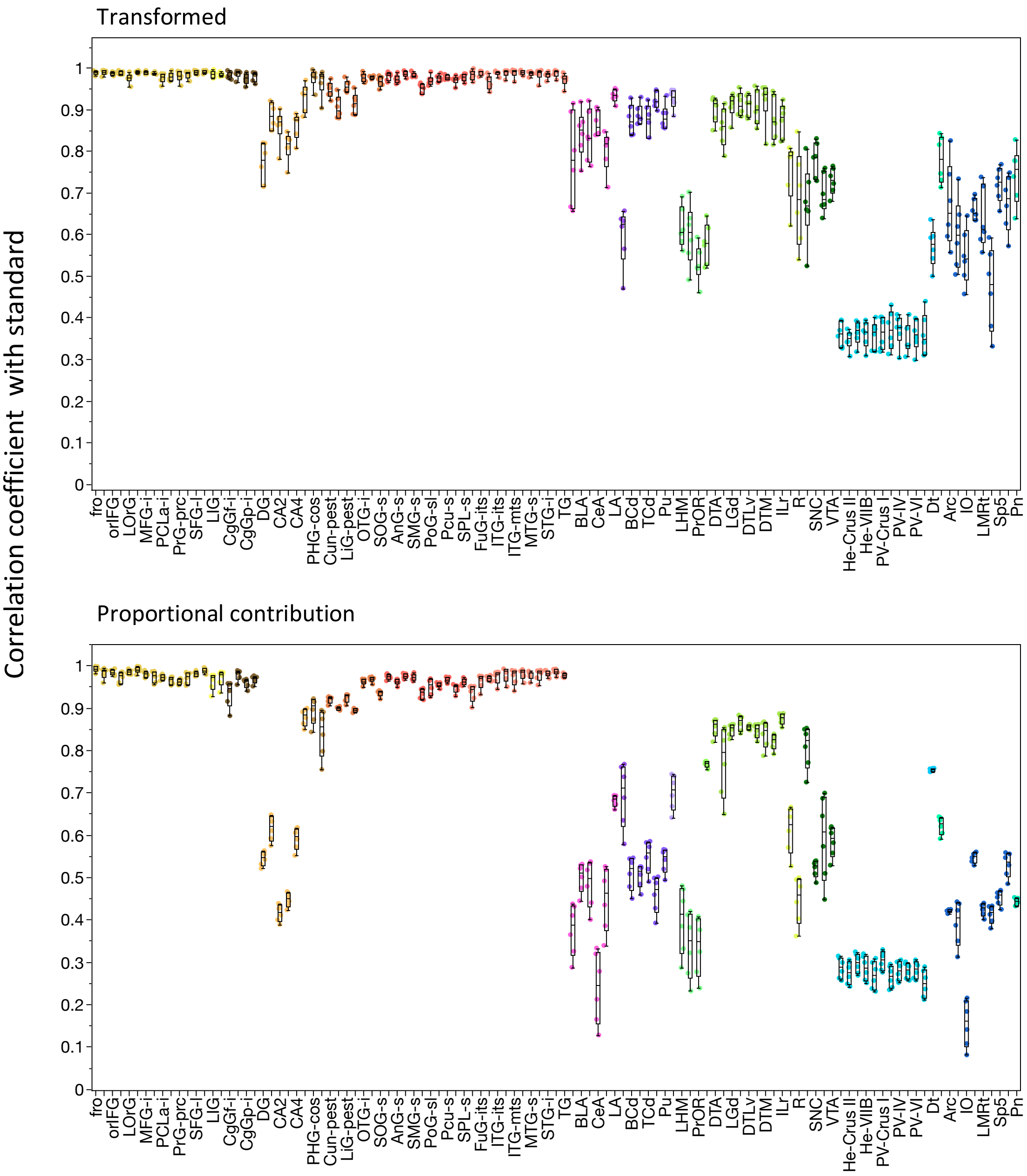

Supplementary Figure 10. Correlation coefficients of Euclidian distances as a metric of variability. Euclidean distances between levels of expression, using Log2 (top) or proportional contribution (bottom), between subunits per structure per subject can be correlated against a standard, in this case the frontal operculum, (fro) to show variability in subunit expression between substructures. Correlation coefficients closer (*R*) to one indicate higher similarity in expression patterns between the structure and the standard. Brain regions are ordered in antero-posterior axis. Each dot is a single subject. The median is represented by the line within the box, and the 1st and 3rd quartiles are represented by the ends of the box. The whiskers extend from each end of the box to the 1st or 3rd quartile ± 1.5 (interquartile range). FL, frontal lobe; Ins, insula; CgG, Cingulate gyrus; HiF, hippocampal formation; PHG, parahippocampal gyrus; OL, occipital lobe; PL, Parietal lobe; TL, temporal lobe; Amg, amigdala; GP, globus pallidus; Str, striatum; Cl, claustrum; Hy, hypothalamus; SbT, subthalamus; DT, dorsal thalamus; VT, ventral thalamus; MES, mesencephalon; CbCx, cerebellar cortex; CbN, cerebellar nuclei; Bpons, basal part of the pons; PTg, pontine tegmentum; MY, myelencephalon. For substructures abbreviations please see Supplementary Table 2.

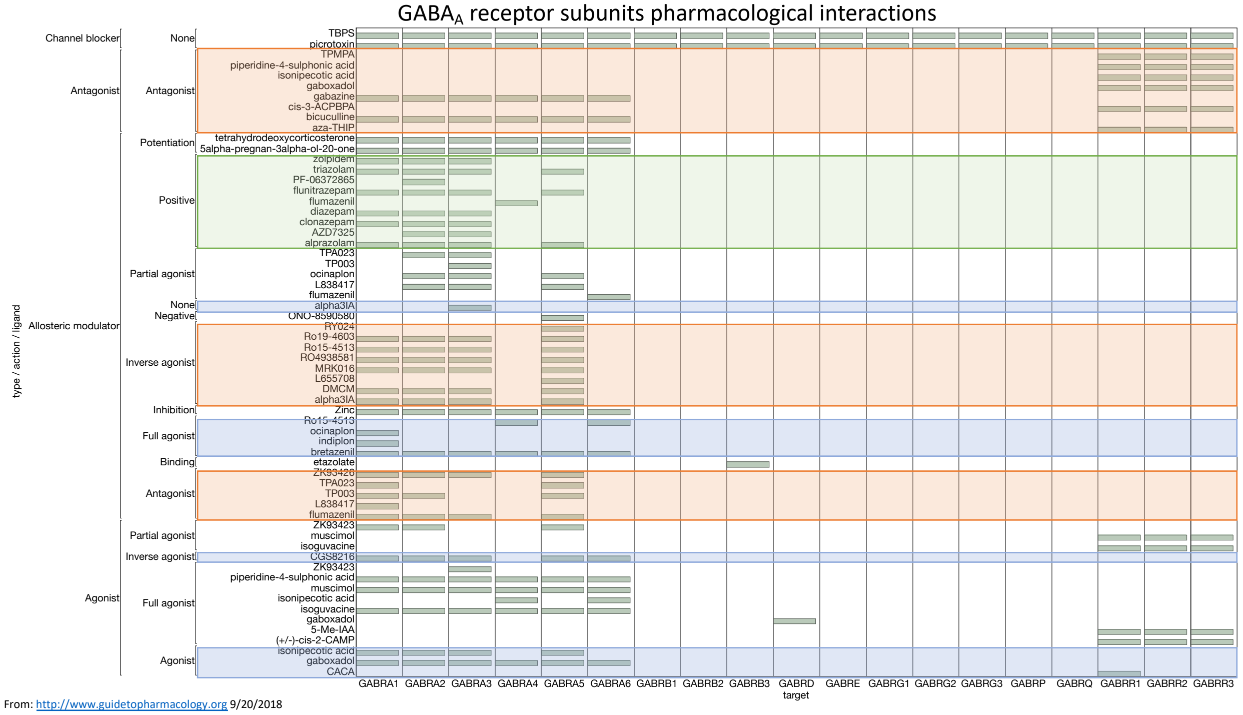

Supplementary Figure 11. Pharmacological interactions of GABA$_A$Rs. Information is based on data downloaded from the International Union of Basic and Clinical Pharmacology on 9/20/2018.
