# Supplementary material for: Human brain transcriptome analysis finds region- and subject-specific expression signatures of GABAAR subunits
Source: Commun Biol. 2019 May 1;2:153. doi: 10.1038/s42003-019-0413-7 (PMC6494906; doi:10.1038/s42003-019-0413-7)
Supplement: Supplementary file 13 — Reporting Summary [file 42003_2019_413_MOESM13_ESM.pdf]

nature research

Corresponding author(s): Adolfo Sequeira and Agenor Limon

Reporting Summary

Nature Research wishes to improve the reproducibility of the work that we publish. This form provides structure for consistency and transparency in reporting. For further information on Nature Research policies, see Authors & Referees and the Editorial Policy Checklist.

Statistical parameters

When statistical analyses are reported, confirm that the following items are present in the relevant location (e.g. figure legend, table legend, main text, or Methods section).

n/a	Confirmed	
☐	☒	The exact sample size (n) for each experimental group/condition, given as a discrete number and unit of measurement
☒	☐	An indication of whether measurements were taken from distinct samples or whether the same sample was measured repeatedly
☐	☒	The statistical test(s) used AND whether they are one- or two-sided<br>*Only common tests should be described solely by name; describe more complex techniques in the Methods section.*
☐	☒	A description of all covariates tested
☐	☒	A description of any assumptions or corrections, such as tests of normality and adjustment for multiple comparisons
☐	☒	A full description of the statistics including central tendency (e.g. means) or other basic estimates (e.g. regression coefficient) AND variation (e.g. standard deviation) or associated estimates of uncertainty (e.g. confidence intervals)
☐	☒	For null hypothesis testing, the test statistic (e.g. F, t, r) with confidence intervals, effect sizes, degrees of freedom and P value noted<br>*Give P values as exact values whenever suitable.*
☒	☐	For Bayesian analysis, information on the choice of priors and Markov chain Monte Carlo settings
☐	☒	For hierarchical and complex designs, identification of the appropriate level for tests and full reporting of outcomes
☐	☒	Estimates of effect sizes (e.g. Cohen's d, Pearson's r), indicating how they were calculated
☐	☒	Clearly defined error bars<br>*State explicitly what error bars represent (e.g. SD, SE, CI)*

Our web collection on statistics for biologists may be useful.

Software and code

Policy information about availability of computer code

Data collection	No software was used for the collection of data
Data analysis	Data analyses and plotting were implemented in JMP version 14 discovery from SAS, the Euclidian distance analysis was implemented in RStudio using R3.5.0 and the mosaic package for R Markdown, QC and SCC were implemented as a custom code of published algorithms using Matlab version 9.1.10.<br>Horn, D. & Gottlieb, A. Algorithm for data clustering in pattern recognition problems based on quantum mechanics. Phys Rev Lett 88, 018702, doi:10.1103/PhysRevLett.88.018702 (2002).<br>Dhillon, I. Co-clustering documents and words using bipartite spectral graph partitioning. . 269–274 (ACM Press, 2001)

For manuscripts utilizing custom algorithms or software that are central to the research but not yet described in published literature, software must be made available to editors/reviewers upon request. We strongly encourage code deposition in a community repository (e.g. GitHub). See the Nature Research guidelines for submitting code & software for further information.

Data

Policy information about availability of data

All manuscripts must include a data availability statement. This statement should provide the following information, where applicable:

- Accession codes, unique identifiers, or web links for publicly available datasets
- A list of figures that have associated raw data
- A description of any restrictions on data availability

The data that support the findings of this study are available from the Atlas Brain Bank (http://human.brain-map.org), the Aging, Dementia and TBI study (http://aging.brain-map.org) and the cell-type Allen study (http://celltypes.brain-map.org/download#transcriptomics)

Field-specific reporting

Please select the best fit for your research. If you are not sure, read the appropriate sections before making your selection.

☒ Life sciences ☐ Behavioural & social sciences ☐ Ecological, evolutionary & environmental sciences

For a reference copy of the document with all sections, see nature.com/authors/policies/ReportingSummary-flat.pdf

Life sciences study design

All studies must disclose on these points even when the disclosure is negative.

Sample size	We download two publicly available datasets of microarray and gene expression of control subjects. The Allen Atlas microarray data has 6 subjects between 24 and 57 years of age with no known neuropsychiatric or neuropathological history. The Aging, Dementia and TBI study has 56 subjects between 78 and 99 years of age with no known neuropsychiatric history. The cell-type study used includes 13,348 individual nuclei from one 54 years old male and one 43 years old, female.
Data exclusions	Since we are interested in population variability we did not exclude any subject. For the microarray analysis we only used brain substructures that were measured in all 6 subjects. We did not investigate the white matter in the micrarray dataset due to large variability and low number of subjects. The cell-type data sets contains 8 subjects, however almost 85% of data comes from one male and one female; we only used these male and female for the study to avoid to include subjects with low sampling in the number of cells.
Replication	All analyses, except quantum clustering (QC) and spectral co-clustering (SCC), were originally done in JMP 14 and then repeated in RStudio using R3.5.0 and the mosaic package for R Markdown with the same results. Unbiased QC and SCC provide similar results to unsupervised hierarchical clustering.
Randomization	No randomization was used in this analysis
Blinding	Blinding was not used in the analysis. However unsupervised and unbiased methods were used in the analyses.

Reporting for specific materials, systems and methods

Materials & experimental systems

n/a	Involved in the study
☒	☐ Unique biological materials
☒	☐ Antibodies
☒	☐ Eukaryotic cell lines
☒	☐ Palaeontology
☒	☐ Animals and other organisms
☒	☐ Human research participants

Methods

n/a	Involved in the study
☒	☐ ChIP-seq
☒	☐ Flow cytometry
☒	☐ MRI-based neuroimaging
